# Highly Efficient CRISPR-Mediated Base Editing in *Sinorhizobium meliloti*

**DOI:** 10.3389/fmicb.2021.686008

**Published:** 2021-06-18

**Authors:** Longxiang Wang, Yuan Xiao, Xiaowei Wei, Jimin Pan, Deqiang Duanmu

**Affiliations:** ^1^State Key Laboratory of Agricultural Microbiology, Huazhong Agricultural University, Wuhan, China; ^2^College of Life Science and Technology, Huazhong Agricultural University, Wuhan, China

**Keywords:** CRISPR/Cas9, *Sinorhizobium meliloti*, base editing, ABEs, CBEs, GBEs, Golden Gate Assembly, multiplex gene editing

## Abstract

Rhizobia are widespread gram-negative soil bacteria and indispensable symbiotic partners of leguminous plants that facilitate the most highly efficient biological nitrogen fixation in nature. Although genetic studies in *Sinorhizobium meliloti* have advanced our understanding of symbiotic nitrogen fixation (SNF), the current methods used for genetic manipulations in *Sinorhizobium meliloti* are time-consuming and labor-intensive. In this study, we report the development of a few precise gene modification tools that utilize the CRISPR/Cas9 system and various deaminases. By fusing the Cas9 nickase to an adenine deaminase, we developed an adenine base editor (ABE) system that facilitated adenine-to-guanine transitions at one-nucleotide resolution without forming double-strand breaks (DSB). We also engineered a cytidine base editor (CBE) and a guanine base editor (GBE) that catalyze cytidine-to-thymine substitutions and cytidine-to-guanine transversions, respectively, by replacing adenine deaminase with cytidine deaminase and other auxiliary enzymes. All of these base editors are amenable to the assembly of multiple synthetic guide RNA (sgRNA) cassettes using Golden Gate Assembly to simultaneously achieve multigene mutations or disruptions. These CRISPR-mediated base editing tools will accelerate the functional genomics study and genome manipulation of rhizobia.

## Introduction

Rhizobia are widespread gram-negative soil bacteria that are mainly classified into two clades, α- and β-proteobacteria. Rhizobia use nitrogenase to directly convert atmospheric nitrogen (N_2_) into ammonium (Masson-Boivin et al., 2009; Seefeldt et al., 2009; Poole et al., 2018). A number of rhizobial species establish symbiotic relationships with their leguminous hosts that drive the most efficient form of nitrogen fixation in nature. Therefore, symbiotic nitrogen fixation (SNF) plays an extremely important role in agriculture (Roy et al., 2020). *Sinorhizobium meliloti* has been reclassified as *Ensifer meliloti* and is one of the well-studied symbiotic nitrogen-fixing bacteria, which nodulates particular plant genera, such as *Medicago truncatula*. The *S. meliloti* genome consists of a chromosome (3.65 Mb) and two megaplasmids, pSymA (1.35 Mb) and pSymB (1.68 Mb). Most of the gene clusters involved in the synthesis of Nod factors (*nod*), nitrogen fixation (*nif*), and nitrogen metabolism are located on pSymA (Galibert et al., 2001).

A variety of approaches have been developed to investigate the unknown function of specific genes, including forward- and reverse-genetic techniques (diCenzo et al., 2019). The homologous recombination-based double crossover system is a routine strategy widely used to generate single gene knock-out mutants (Quandt and Hynes, 1993; Schafer et al., 1994). Site-specific recombinase systems are also powerful techniques for producing large-scale genome deletions and gene insertions in *S. meliloti*. These systems include the Flp/*FRT* (Milunovic et al., 2014), Cre/*loxP* (Harrison et al., 2011), ΦC31 integrase (Heil et al., 2012), and lambda integrase (House et al., 2004) systems. Tn5-based transposon mutagenesis is the most important technique in the history of rhizobial genetic and genomic research. As a powerful forward-genetic analysis method, Tn5-based transposon mutagenesis is suitable for generating a larger number of mutants. Indeed, a few key nodulation and nitrogen fixation genes were identified using this method (Long et al., 1988; Arnold et al., 2017; diCenzo et al., 2018). Although these methods have fundamentally contributed to rhizobial functional genomics research, they are time-consuming and labor-intensive. Thus, there is a critical need for the development of alternative approaches for genome manipulation in rhizobia.

The clustered regularly interspaced short palindromic repeats (CRISPR) and CRISPR-associated (Cas) systems derived from bacterial and archaeal immune systems have been repurposed for precise genome editing (Cong et al., 2013; Mali et al., 2013). This powerful CRISPR/Cas9 system is comprised of a CRISPR-associated protein 9 nuclease (Cas9) and an engineered single-guide RNA (sgRNA) containing a specific 20 nt protospacer that specifies the target site in the genome. With the guidance of the sgRNA, the Cas9/sgRNA complex targets the specific genomic region located next to the protospacer adjacent motif (PAM) and induces double-strand breaks (DSBs) in the genomic DNA. Subsequently, cells use homologous recombination (HR) or the non-homologous end joining (NHEJ) pathway to rapidly repair the DSBs, which creates either insertions or deletions (indels) and thus, disrupts target genes. CRISPR-Cas9 mediated genome engineering has been reported in a variety of organisms, including *Escherichia coli* (Jiang et al., 2013), higher plants (Shan et al., 2013), and mammalian cells (Cong et al., 2013). Unlike animals and higher plants, bacteria mainly rely on HR rather than the NHEJ pathway to repair DSBs. Thus, to generate a bacterial mutant, a donor DNA template harboring the desired changes (e.g., point mutations or indels) must be provided. This complex procedure for clone construction limits the application of this novel technique.

Recently, several groups reported a new approach for the site-directed mutagenesis of genomic DNA that is called the “base editing” system. These approaches utilize a Cas9 nickase fused to various deaminases. These fusion proteins catalyze specific C-to-T or A-to-G transitions in genomic DNA. The cytidine base editors (CBEs) mainly include two representative architectures, the BE (base editor) (Komor et al., 2016) and Target-AID (target-activation-induced cytidine deaminase) systems (Nishida et al., 2016). Cytidine deaminase catalyzes the deamination of targeted C to yield uracil (U). U is recognized as T and thus, leads to the transition of the original C-G to a T-A base pair following DNA repair or replication. UGI (uracil-DNA glycosylase inhibitor protein) is an inhibitor of nucleotide excision repair (NER) that increases the C-to-T conversion efficiency. Due to the catalytic activity of various deaminases and the architectures of the base editors, the deamination can occur in different regions of the protospacer, which is referred to as the “editing window.” The adenosine base editors (ABEs) consist of a heterodimeric TadA (TadA-TadA^∗^) constructed with an artificially evolved DNA adenine deaminase (TadA^∗^) derived from *E. coli* tRNA adenosine deaminase (TadA) and wild type (TadA), an XTEN linker, and the Cas9 nickase (Gaudelli et al., 2017). The adenine deaminase catalyzes the deamination of A to inosine (I) in the target region. During the following DNA repair or replication, I is recognized as G resulting in the transition of the original A-T to a G-C base pair. The base editing systems are highly efficient, lower the rate of off-target effects, and do not require either DNA double-strand cleavage or the repair of a donor template. These dramatic improvements have made base editing a widely applicable tool for gene disruption in a variety of bacteria (Banno et al., 2018; Gu et al., 2018; Zheng et al., 2018; Tong et al., 2019; Rodrigues et al., 2021).

In this study, we engineered single-plasmid CRISPR-mediated base editing tools for *S. meliloti*, containing adenosine base editors (ABEs), cytidine base editors (CBEs), and glycosylase base editors (GBEs) that could efficiently achieve both base transitions (A-to-G, C-to-T) and transversions (C-to-G). These base editors are amenable to the assembly of multiplex sgRNAs cassettes using Golden Gate assembly to simultaneously achieve multigene mutations or disruptions. A whole-genome prediction of guide RNAs for gene inactivation indicates that nearly 88% of the possible editable stop codons are located in the first half of the gene coding regions in the *S. meliloti* genome. As an effective supplement to the current double-crossover gene knockout technique, the Tn5-based transposon system, and the site-specific recombinases system, the establishment and application of these CRISPR-mediated base editing tools will facilitate the functional genomic studies and genome manipulation in rhizobia.

## Materials and Methods

### Bacterial Strains and Culture Conditions

The constructed plasmids were used to transform *E. coli Trans* 1-T1(F^–^ φ80 (*lac*Z) ΔM15 Δ*lac*X74 *hsd*R (r_k_^–^, m_k_^+^) Δ*rec*A1398 *end*A1 *ton*A; TransGen Biotech Co., Ltd, China) or *Trans* DB3.1 (F^–^ *gyr*A462 *endA*1 Δ(*sr*1-*rec*A) *mcr*B *mrr hsd*S20(r_B_^–^, m_B_^–^) *sup*E44*ara*-14 *gal*K2 *lac*Y1 *pro*A2 *rps*L20 (Sm^R^) *xy*l-5 λ- *leu mtl*1). *E. coli* cells were grown aerobically at 37°C in Lysogeny Broth (LB) (Bertani, 1951) that was supplemented with gentamicin (50 μg mL^–1^), kanamycin (50 μg mL^–1^), or chloramphenicol (50 μg mL^–1^). Rhizobial cells (*S. meliloti* strain 1,021) were cultured with tryptone yeast (TY) medium (Beringer, 1974) supplemented with fosfomycin (50 μg mL^–1^) and neomycin (50 μg mL^–1^). Rhizobial strains harboring plasmids that contain the *pTau* promoter were grown in a medium that also contained 40 μM taurine, which served as an inducer.

### Construction of Base Editing Plasmids

The backbone of the plasmid pGm was constructed in two steps. First, the pre-plasmid was constructed with four different elements derived from pOGG004, pOGG009, pOGG011, and pOGG013 (Geddes et al., 2018) using a type IIS restriction enzyme-based Golden Gate Assembly (GGA) (Engler et al., 2008, 2009). Next, the pre-plasmid was digested with *Bsa*I and the multiple cloning site region was replaced with a DNA fragment produced by annealing the KX-oligo-F and the KX-oligo-R. This DNA fragment harbors only *Kpn*I and *Xba*I restriction sites. To construct the pKm plasmid, the gentamicin-resistance gene in the pGm pre-plasmid was replaced with a neomycin-resistance gene that was amplified from pOPS0314 (Geddes et al., 2018) using the Kan-*Esp*3I-F and Kan-*Esp*3I-R primers. Then the multiple cloning site in the pKm pre-plasmid was replaced with a DNA fragment generated by annealing the KX-oligo-F and KX-oligo-R oligonucleotides. These primers and oligonucleotides are described in Supplementary Table 3.

The genes that encode a codon-optimized Cas9 nickase named Cas9n-D10A, cytidine deaminase named APOBEC1 from rat, activation-induced cytidine deaminase (AID) ortholog named PmCDA1, uracil DNA glycosylase inhibitor named UGI, adenine deaminase dimer named ABE7.10, and uracil-DNA glycosylase named UNG were synthesized by GenScript Biotech (Nanjing, China). The expression of the codon optimized Cas9n and fusion proteins containing this codon optimized Cas9n were designed to be driven by three different promoters, the δ-aminolevulinic acid synthetase gene promoter (*pHemA*) amplified from *S. meliloti* genomic DNA (Leong et al., 1985), the constitutive neomycin cassette promoter (*pNeo*) amplified from plasmid pOPS0314 (Geddes et al., 2018), and the taurine-inducible promoter (*pTau*) amplified from plasmid pOPS0359 (Geddes et al., 2018).

The expression of the synthetic guide RNA (sgRNA) cassette was designed to be driven by five different promoters, the housekeeping sigma factor σ^70^ gene promoter (*pSigA*) (Ramirez-Romero et al., 2006), the sigma factor σ^54^ gene promoter (*pRpoN*) (Clark et al., 2001), the *pRpmJ* and *pRpsT* promoters from genes that encode ribosomal proteins, and the Tyr-tRNA (GTA) gene promoter (*pTyr*) (MacLellan et al., 2006). The two expression cassettes, Cas9n fusion proteins and sgRNA, are transcribed in a head-to-tail pattern and separated by the *rrnBT1* terminator. The entire expression cassettes were first constructed in the pBluescript SK^+^ plasmid. Then the pre-plasmid was digested with *Kpn*I and *Xba*I and inserted into the pKm vectors. All constructed plasmids were verified by restriction enzyme digestion and Sanger sequencing.

The 20-nt long guide RNAs were designed using BE-designer^1^, which is an online design tool for CRISPR base editing (Hwang et al., 2018). The synthetic oligonucleotides were annealed and ligated into linearized target plasmids. The products of the ligation reactions were used to transform *E. coli* competent cells. The positive clones were identified and analyzed using colony PCR and Sanger sequencing. All of the primers used in the clone construction are listed in Supplementary Table 3. All of the DNA sequences are listed in Supplementary Table 4. The four vectors are available from Addgene (Addgene ID: pKm, 170331; pKm-HABE, 170332; pKm-HCBE, 170333; pKm-HGBE, 170334).

### Preparation of Competent Cells, Electroporation, and Base Editing

As the recipient strain, *S. meliloti* strain 1021 was prepared for electroporation based on a published procedure with minor modifications (Ferri et al., 2010). A single colony was picked from 2-day-old colonies on solid TY medium and grown in liquid TY medium as the seed strain for more than 16 h. The seed strain was inoculated (2% v/v) into 250 mL of fresh TY medium and grown for another 6–8 h until the OD_600_ reached ∼0.6. Cells were chilled on ice for 30 min and harvested by centrifugation at 3,000 × *g* for 20 min at 4°C. The pellet was washed twice with 30–50 mL of glycerol (10% v/v) and resuspended in 3 mL of glycerol (10% v/v). From this, 50 μL of competent cells were aliquoted into separate tubes and stored at −80°C. To these competent cells, 3–5 μL of plasmid (200 ng/μL) was added. Electroporation was performed using a BTX ECM 630 electroporator with the following parameters: voltage 2,300 v, resistance 200 Ω, capacitance 25 μF, the time constant 4.8 ms, and cuvette gap width of 1 mm. Then, these cells were immediately suspended in 1.0 mL of ice-cold TY (containing 10% v/v glycerol) medium and incubated for 3 h at 28°C on an orbital shaker at 220 rpm. For plasmids harboring *pTau*, after electroporation, the cells were immediately suspended in the same medium containing 40 μM taurine, which serves as an inducer. The cells were centrifuged for 15 s at 13,500 × *g* and plated on a solid TY medium containing appropriate antibiotics and inducers. After 5–7 days, eight individual colonies were picked from each plate and were genotyped using a colony PCR procedure that utilized primers that span all designed target sites of the genes. The colonies were independently analyzed using Sanger sequencing. All of the primers used for the genotyping are listed in Supplementary Table 3.

### Plant Growth, Inoculation, and Phenotypic Assay

*Medicago truncatula* ecotype Jemalong A17 was used for the SNF phenotypic assay. Seed sterilization and germination were performed according to the protocol described by Wang et al. (2016) with minor modifications. In brief, seeds were surface sterilized and germinated on MS (Murashige and Skoog) medium. The plates were placed upside-down to promote hypocotyl elongation at 21°C in the dark for 24 h. Seedings were then transferred to nutrition pots filled with vermiculite and perlite (3:1) and grown in a greenhouse at 21°C in a photoperiod regime containing 16 h of light and 8 h of dark. After 3–5 days of growth, plants were inoculated with either *Sm1021* (wild type) or *Sm1021-nodA* (W7^∗^ mutant) or else were not inoculated. At 28 d post inoculation, plants were harvested for the symbiotic phenotype assay.

### GGA-Based Multiplex Gene Base Editing

Plasmids used for the multiplex precise base editing were constructed using the type IIS restriction enzymes-based Golden Gate Assembly (GGA) (Engler et al., 2008, 2009). The DNA oligonucleotides harboring *Bsa*I enzyme sites and different overhangs were designed using GETSET^TM^, which is a web-based tool from NEB^2^. For example, the plasmid (pKm-HCBE2-*nodABC*) harboring three different guide RNAs targeting *nodA*, *nodB*, and *nodC* was constructed using the GGA with the *Bsa*I-linearized acceptor vector pKm-HCBE2 and three PCR products amplified from pKm-HCBE2-*nodA* (part1, linker1-*nodA*sgRNA-ter-linker2), pKm-HCBE2-*nodB* (part2, linker2-*pRpmJ*:*nodB*sgRNA-ter-linker3), and pKm-HCBE2-*nodC* (part3, linker3-*pRpmJ*:*nodC*sgRNA-linker4). All of the PCR products were amplified using KOD-PLUS-NEO polymerase (TOYOBO, Japan) and gel purified with a SiO_2_-based gel extraction kit. Primers for plasmid construction are listed in Supplementary Table 3.

### PCR-Based Off-Targets Assay

The prediction and analysis of off-target sites for the *nodA* locus was performed using Cas-OFFinder^3^. In total, eight loci with the greatest sequence similarity relative to the target locus were chosen as putative off-target sites, and 10 colonies were selected and grown in 5 mL of liquid TY medium at 30°C for two d. A 2 mL aliquot of culture was harvested for genomic DNA extraction using a bacterial genomic DNA kit (TransGen Biotech, Beijing). Mutations in the off-target sites were analyzed using PCR and Sanger sequencing. The primers used for off-target assay are listed in Supplementary Table 3.

### Plasmid Curing

After gene modification, plasmid curing was performed. The sucrose-induced *sacB* gene from *Bacillus subtilis* was used as a counter-selectable marker for the construction of suicide vectors in rhizobium (Quandt and Hynes, 1993; Schafer et al., 1994). The *sacB* gene was amplified from pK18mobsacB (Schafer et al., 1994), inserted into the *Xba*I site of the base editing vectors using Gibson assembly, and used for plasmid curing.

The colonies of positive mutants were picked and grown in 5 mL of liquid TY medium at 30°C for 2 days. These growth conditions lead to partial plasmid loss in cells without the selection pressure provided by antibiotics. The cell culture was diluted 10^4^ fold with a fresh TY medium. A 100 μL aliquot of diluted cells was spread on a TY plate supplemented with 10% sucrose for 3–4 days. The colonies that survived were grown in a liquid TY medium and then spread on a TY medium supplemented with or without appropriate antibiotics to test for plasmid curing.

### Analysis of Guide RNAs for Potential Genome-Wide Gene Inactivation

The cytidine base editors targeting CGA (Arg), CAG (Gln), and CAA (Gln) in the coding strand and CCA in the non-coding strand could create TGA, TAG, and TAA stop codons, respectively, by catalyzing C-to-T transitions. Positions 1–8, 12, and 15 of the 20 nt protospacer are hotspots for the CBE2 system. Counting the protospacer adjacent motif (PAM) as positions 21–23, we performed a whole-genome analysis of guide RNA target sites to identify their potential for gene inactivation. The assay was performed using the iSTOP procedure (Billon et al., 2017) with minor modifications and Circos^4^ (Krzywinski et al., 2009) was used for visualizing editable genes of the CBE systems in the *S. meliloti* genome.

## Results

### Design of a Modularized Single-Plasmid Adenosine Base Editing Tool for *Sinorhizobium meliloti*

**FIGURE 1:**
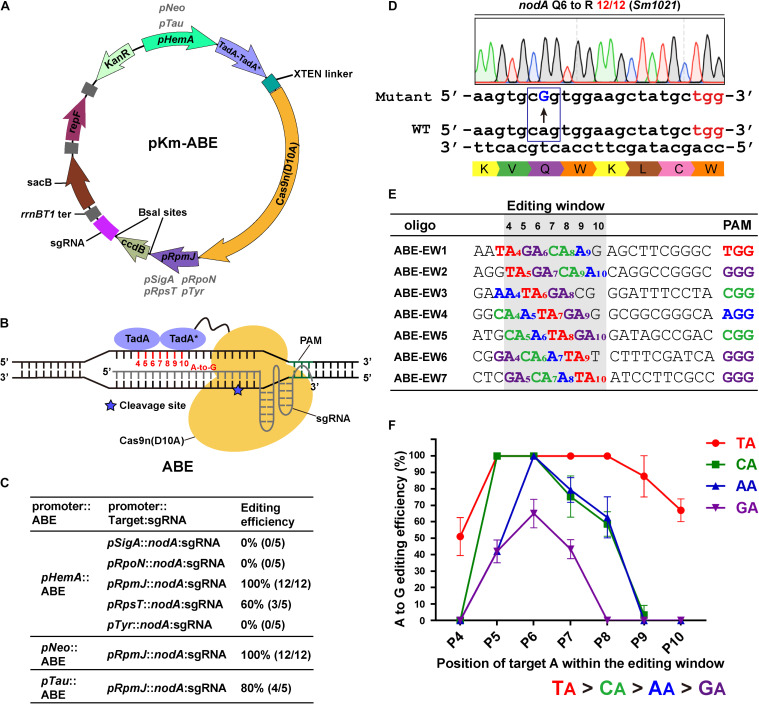
Adenine base editor (ABE) systems catalyze efficient A-to-G conversions in *Sinorhizobium meliloti*. **(A)** Map of the pKm-ABE plasmid. KanR: the kanamycin-resistant marker for selection in *E. coli* and the neomycin-resistant marker for selection in *S. meliloti*; repF: pBBR1 origin of replication and oriT from *E. coli*; rrnBT1 ter: rrnBT1 transcriptional terminator; *sacB*: the counter-selectable marker for plasmid curing after editing; *pHemA*: the δ-aminolevulinic acid synthetase gene promoter of *S. meliloti*; *pNeo*: the constitutive neomycin cassette promoter; *pTau*: the taurine-inducible promoter of *S. meliloti*; TadA-TadA*-XTEN linker-Cas9n: the Cas9-adenine deaminase fusion protein; *pSigA*: the housekeeping sigma factor σ^70^ gene promoter; *pRpoN*: the sigma factor σ^54^ gene promoter; *pRpsT* and *pRpmJ*: the ribosomal protein-related gene promoters; *pTyr*: the Tyr-tRNA (GTA) gene promoter; *ccdB*: a *Bsa*I-flanking *ccdB* cassette for spacer sequence insertions. **(B)** Schematic diagram of Adenine Base Editors (ABEs). TadA-TadA*: artificially evolved DNA adenine deaminase derived from *E. coli* tRNA adenosine deaminase (TadA). TadA*: the mutated TadA. The SpCas9 nickase (Cas9n D10A) is fused to the C-terminus of the TadA-TadA* and XTEN linker cassettes. The estimated activity window of ABE systems ranges from position 4 to 10 of the protospacer. The protospacer adjacent motif (PAM) ranges from position 21 to 23. The asterisk indicates the cleavage site of Cas9n nickase. **(C)** Editing efficiency comparison of ABEs and sgRNAs expressed from various promoters. **(D)** Representative A-to-G base editing with an ABE system harboring *pHemA*:ABE and *pRpmJ*:*nodA* expression cassettes. The codon encoding Q6 of the *nodA* gene in the *S. meliloti* was successfully mutated to a codon encoding R with an efficiency of 12/12. The mutated sites are colored blue. The PAM sites are colored red. **(E)** Seven oligonucleotides designed to investigate position effects and optimal NA combinations on editing efficiency of ABE systems. Each NA combination varied from position 4 to 10 within the protospacer. The editing window is shaded in light gray. **(F)** A-to-G editing efficiencies of various NA combinations and target A positions in the editing window. Values and error bars are the mean and SD of three independent biological replicates.

To harness the CRISPR-Cas9 system for genome editing in rhizobium, we designed and constructed a single-plasmid adenosine base editor (ABE) system (Figure 1A). The broad-host-range vector pKm was generated using a Golden Gate Assembly (GGA) approach to serve as the backbone plasmid. The ABE consists of a heterodimeric TadA (TadA-TadA^∗^) fused to the N-terminus of the Cas9 nickase with an XTEN linker (Figures 1A,B; Gaudelli et al., 2017) that was codon-optimized and synthesized. The ABEs and the sgRNAs are transcribed in a head-to-tail pattern and separated by the *rrnBT1* terminator (Figure 1A). One striking feature of this base editing system is the presence of two negative selectable markers (Figure 1A). Two *Bsa*I sites and the entire Cm-*ccdB* cassette (Hecker et al., 2015) were used for one-step seamless cloning of a 20-bp spacer and the *sacB* gene from *Bacillus subtilis*, which was used for plasmid curing after editing (Schafer et al., 1994).

To extend the scope of the base editing application, three routinely used promoters were selected to drive the expression of the ABEs, the δ-aminolevulinic acid synthetase gene promoter (*pHemA*) (Leong et al., 1985), the constitutive neomycin cassette promoter (*pNeo*) (Geddes et al., 2018), and the taurine-inducible promoter (*pTau*) (Geddes et al., 2018). Expression of the fusion proteins could be detected in the host strain *S. meliloti* (Supplementary Figure 1A). The transcription start sites of these routinely used promoters have not been well-studied and therefore, they may add extra nucleotides at the 5′ end of single-guide RNAs (sgRNAs). These extra nucleotides could interfere with the editing efficiency and mislead the activity window analysis. Therefore, to develop a system that accurately expresses sgRNAs, we systematically tested and selected five promoters, including the housekeeping sigma factor σ^70^ *sigA* promoter (*pSigA*) (Ramirez-Romero et al., 2006), the sigma factor σ^54^ *rpoN* promoter (*pRpoN*) (Clark et al., 2001), the ribosomal protein-related *rpsT* and *rpmJ* promoters (*pRpsT* and *pRpmJ*) and the Tyr-tRNA (GTA) promoter (*pTyr*) (MacLellan et al., 2006; Figure 1B).

For a proof of concept, we selected the Nod factor biosynthetic gene *nodA* as a target. We designed a 20-nt gRNA spacer containing potentially editable A(s) within the editable window using BE-Designer (Hwang et al., 2018) and expressed this gRNA using the promoters from *pSigA*, *pRpoN, pRpsT*, *pRpmJ*, or *pTyr*. These five individual constructs were introduced into *S. meliloti*. In total, 5–12 colonies from each plate were picked for colony-PCR and Sanger sequencing after 7 day of growth at 28°C. The editing efficiency was calculated as the percentage of successfully edited events relative to the total number of analyzed colonies. The A at position 7 was changed to G with 60% (*pRpsT*) and 100% (*pRpmJ*) efficiencies, resulting in the substitution of Q6 (CAG) with an R (CGG) residue in the protein encoded by *nodA* (Figures 1C,D). However, *pSigA*, *pRpoN*, and *pTyr-*driven *nodA-*sgRNAs failed to mediate the A-to-G transition. *pHemA*, *pNeo*, and *pTau* efficiently drove the expression of the ABE fusion protein. Therefore, we further investigated the variation in their editing efficiency using the *pRpmJ* promoter to drive the expression of the identical *nodA*-sgRNA. Consistently, we observed A to G transitions with efficiencies of 100% (*pHemA*), 100% (*pNeo*), and 80% (*pTau*) using these promoters (Figure 1C).

We then designed several guide RNA oligonucleotides that target *nodA*, *nodB*, *nodC*, *nifH*, *nifD*, and *nifK* to evaluate the editing efficiencies among the various targets (Supplementary Figures 1B–J). The editing efficiencies exhibited quite huge differences that ranged from 0 to nearly 100%. We also noticed that when multiple As were present in a target region, the editing efficiencies were different (Supplementary Figures 1F,J). To evaluate the effects of the target A position within the editing window and the guide RNA sequence context on editing efficiency, we designed seven guide RNAs containing all four possible NA combinations (N = A, G, C, or T) covering the potential editing window from position 4 to 10 (Figure 1E; Tong et al., 2019). By calculating the A-to-G conversion efficiency, we found that the ABE system catalyzed the deamination reaction with the following preference: TA > CA > AA > GA (Figure 1F). Although the order of priority was not quite consistent with the *in vivo* results reported previously (Tong et al., 2019), the TA combination was the most efficiently edited in both *S. meliloti* and *Streptomyces coelicolor* (Tong et al., 2019). Moreover, we found that the editing window spanned from position 4 to position 10 (Figures 1A,F), which is a wider window than reported previously (Gaudelli et al., 2017; Tong et al., 2019; Zhang Y. et al., 2020) and that positions 5–8 were edited with the highest efficiencies (Figure 1F).

To evaluate the potential off-target effects of the ABE system in *S. meliloti*, we selected the top eight loci containing the most similar sequences to *nodA* (oligo 2) using Cas-OFFinder (Bae et al., 2014) and analyzed these loci using a PCR-based sequencing strategy. No notable off-target events were observed (Supplementary Table 1). These data provide evidence that the ABE system is highly accurate, which is consistent with previous reports (Zhang Y. et al., 2020).

### Cytidine Base Editors Catalyze Accurate C-to-T Transitions in *S. meliloti*

**FIGURE 2:**
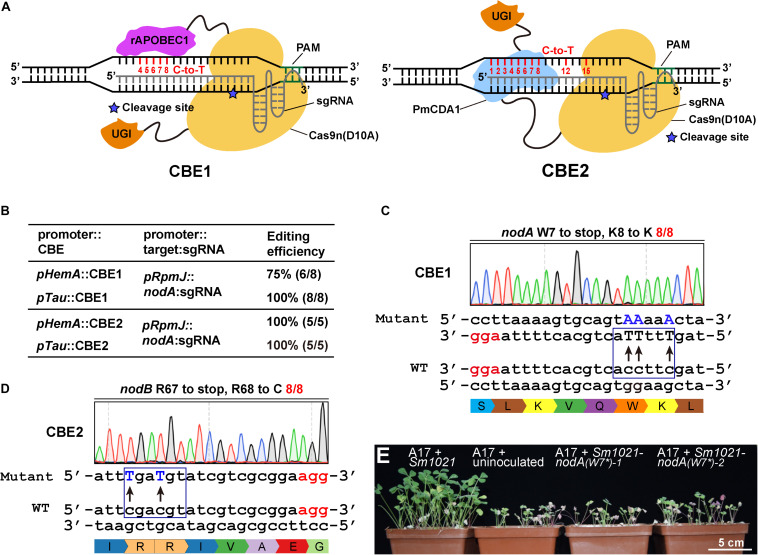
Cytidine base editor (CBE) systems enable efficient C-to-T conversion in *S. meliloti*. **(A)** Schematic diagram of two typical cytidine base editors (CBEs). The CBE1 base editor contains the rat APOBEC1(rAPOBEC1) cytidine deaminase and the Uracil DNA glycosylase inhibitor (UGI) fused to the N-terminus and C-terminus of the SpCas9 nickase (Cas9n D10A), respectively. The CBE2 base editor contains the activation-induced cytidine deaminase (AID) ortholog PmCDA1 and UGI proteins fused to the C-terminus of the Cas9 nickase. The potential editable sites of the CBE systems are highlighted in red. The asterisk indicates the cleavage site of Cas9n nickase. **(B)** Editing efficiency comparison of various combinations of CBEs, promoters, and sgRNAs. **(C)** Conversion of the codon encoding the W7 residue of the *nodA* gene in *S. meliloti* to a stop codon. The conversion efficiency was 8/8. The mutated sites are colored blue. The PAM sites are colored red. **(D)** Conversion of the codons encoding R67 and R68 in the *nodA* gene in *S. meliloti* to stop codons and codons encoding R. The conversion efficiency was 12/12. **(E)** Growth phenotype of *Medicago truncatula*. Under nitrogen limiting conditions, host plants exhibited robust vegetative growth 4 weeks after they were inoculated with wild type *Sm1021.* In contrast, growth was retarded in plants that were inoculated with Sm1021-nodA(W7*) and in plants that were not inoculated. Two mutant bacterial strains were generated using the *pTau*:CBE1 system as shown in panel C.

Cytidine base editors (CBEs) (Komor et al., 2016; Nishida et al., 2016) have been widely utilized for catalyzing precise cytidine-to-thymine transitions in a variety of bacterial species. Two representative cytidine base editors were chosen to assess the possibility of cytidine base editing in *S. meliloti*. The CBE1 base editor contains the rat APOBEC1 (rAPOBEC1) cytidine deaminase and UGI fused to the N-terminus and C-terminus of the Cas9 nickase (Figure 2A). The CBE2 base editor contains the PmCDA1 cytidine deaminase and UGI fused to the C-terminus of the Cas9 nickase (Figure 2A).

To extend the scope of the base editing application, we chose to evaluate the efficiencies of CBEs that were expressed from *pHemA-* and *pTau*. For these CBEs, the sgRNAs were expressed using identical *pRpmJ* promoters. The editing efficiency of CBE1 expressed from *pTau* was nearly 100%. However, the editing efficiency of CBE1 expressed from *pHemA* was only 75% (Figure 2B). In contrast, the editing efficiencies CBE2 expressed from either *pHemA* or *pTau* were 100% (Figure 2B). Although the editing efficiencies of the CBEs expressed from *pTau* and *pHemA* were equal, *pHemA* is much shorter than *pTau* and does not need taurine as an inducer. We then chose the *pHemA-*based CBE2 system (pKm-HCBE) for follow-up studies. The codon encoding W7 in the *nodA* gene from *S. meliloti* was successfully mutated to a stop codon with an efficiency of 100% (8/8) using the CBE1 system (Figure 2C). The codons encoding R67 and R68 in the *nodB* gene were successfully mutated to stop codons and cytosine (C) with an efficiency of 100% (8/8) using the CBE2 system (Figure 2D). Two *Sm1021-nodA(W7^∗^)* mutants generated using the CBE1 system were chosen for the symbiotic phenotype assay. In nitrogen-limiting conditions, host plants exhibited robust vegetative growth beginning at 4 weeks after inoculation with wild type *Sm1021* relative to plants inoculated with *Sm1021-nodA(W7^∗^)* and uninoculated plants (Figure 2E).

Next, in order to systematically evaluate the editing window of the CBEs, we designed several guide RNA oligonucleotides that target *nodA*, *nodB*, *nodC*, *nifH*, and *nifK* to evaluate the editing efficiency among various targets (Supplementary Figures 2A–H). The editing was almost 100% efficient at positions 1–8 of the protospacer. The PAM was located from position 21 to 23. The editing events also occurred at position 12 and position 15 with efficiencies ranging from 1/8 to 3/8 (Supplementary Figures 2B,D,F,G). In conclusion, the estimated activity window of the CBE systems ranged from position 1 to 8 and also included positions 12 and 15 of the protospacer, which was much broader than reported in previous studies (Chen et al., 2018; Gu et al., 2018; Tong et al., 2019). In addition, no off-target mutations were observed in the eight potential off-target loci (Supplementary Table 2), which indicates a high fidelity for these CBE systems, which is consistent with previous studies (Tong et al., 2019).

### Glycosylase Base Editors Efficiently Convert Targeted Cs Into Gs

**FIGURE 3:**
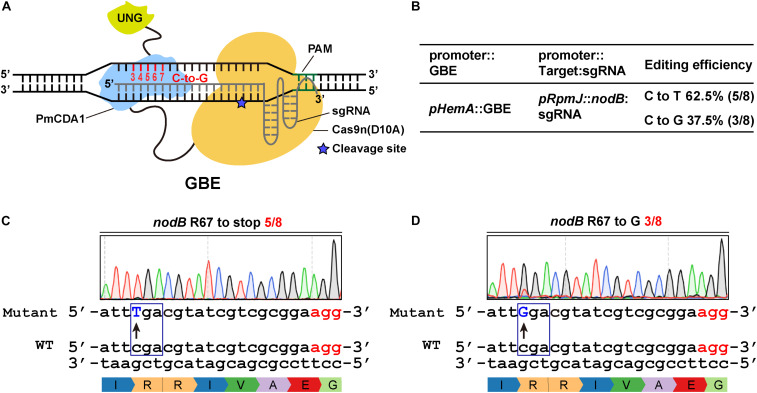
Efficient C-to-G transversions catalyzed by the glycosylase base editor (GBE) system in *S. meliloti*. **(A)** Schematic diagram of a glycosylase base editor (GBE). The activation-induced cytidine deaminase (AID) ortholog PmCDA1 was fused to the C-terminus of the Cas9 nickase. UGI was replaced with the uracil DNA N-glycosylase (UNG), which was fused to the C-terminus of PmCDA1. The potential editable sites of the GBE system are highlighted in red. The asterisk indicates the cleavage site of the Cas9n nickase. **(B)** Editing efficiency of the GBE system. The expression of the GBE fusion protein was driven by the *HemA* promoter. The expression of the guide RNAs was driven by the *RpmJ* promoter. **(C,D)** Conversion of the codon encoding R67 in the *nodB* gene to either a stop codon **(C)** or codon encoding glycine **(D)**. The conversion efficiencies were 5/8 **(C)** and 3/8 **(D)**.

Previous studies indicate that CBEs can catalyze the conversion of C to either A or G (Komor et al., 2016; Nishida et al., 2016). Recently, Kurt et al. (2020) and Zhao et al. (2021) reported novel DNA base transversion tools-glycosylase base editors (GBEs) that catalyze C-to-A and/or C-to-G base conversions. Meanwhile, we found that the CBE1 system could produce a C-to-G mutation rather than a C-to-T mutation when UGI was absent (Supplementary Figures 3A,B). To extend the scope of glycosylase base editors, we replaced UGI, by fusing UNG to the C-terminus of Cas9n-AID (Figure 3A). The expression of the GBE fusion protein was driven by *pHemA*. The expression of the guide RNAs was driven by the *pRpmJ* promoter. The editing efficiency at the target loci was 8/8 and the C-to-G transversion was 3/8 (Figure 3B). The codon encoding R67 in the *nodB* gene in *S. meliloti* was successfully mutated to stop codons and codons encoding glycine (G) with efficiencies of 5/8 and 3/8, respectively (Figures 3C,D). In addition, we designed several guide RNA oligonucleotides targeting nodA, nodC, and nifK to evaluate the editing efficiencies of the various targets (Supplementary Figures 3C–F). The editing efficiency at the target loci and the C-to-G transversion ratio ranged from 66 to 80% and from 33 to 80%, respectively (Figure 3B and Supplementary Figures 3C–F).

### GGA-Based Multiplex Gene Base Editing in *S. meliloti*

Given the simplicity and high efficiency of base editors, researchers working on microbes have adopted versatile tools for accelerating the generation of not only single gene disruption but also multiplexed modifications of gene families or genes involved in biosynthetic pathways (Tong et al., 2019; Zhang Y. et al., 2020). Two approaches were used to express and process sgRNAs for multiplex applications. One approach was stacking multiple cassettes with the same or different combinations of promoters and terminators (Feng et al., 2018). The other strategy was to express polycistronic transcripts containing gRNAs and process them using the Csy4 endoribonuclease (endo-RNase) (Tong et al., 2019). Considering the dramatic decrease in transformation efficiency that occurs when vector length increases, we chose to stack multiple cassettes to generate multiplex mutations in *S. meliloti*.

**FIGURE 4:**
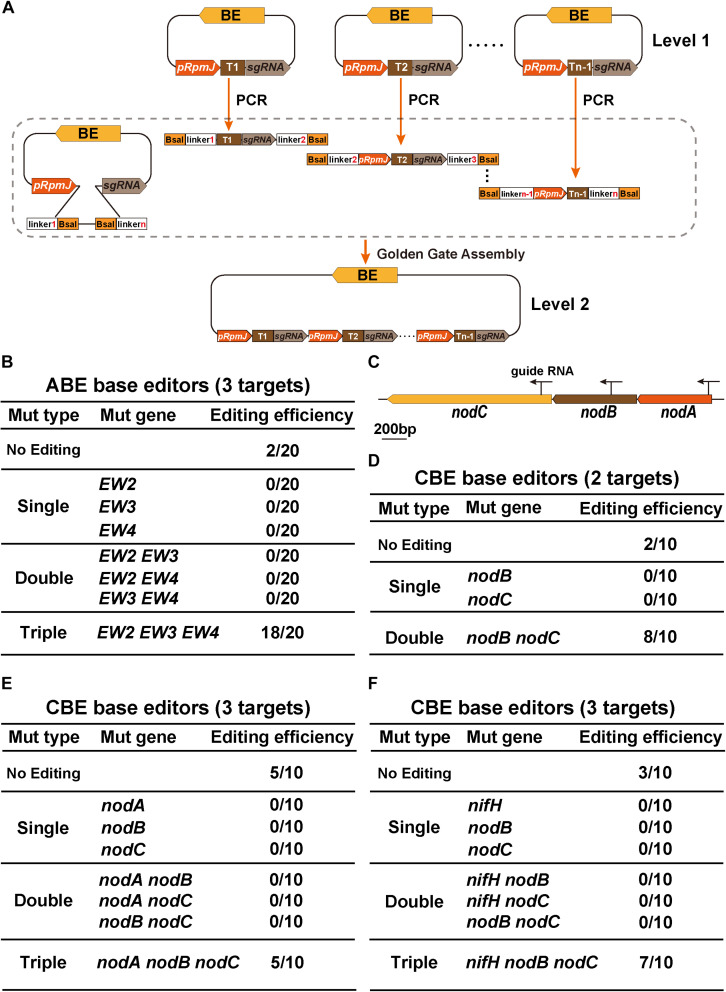
GGA-based multiplex gene editing. **(A)** Schematic flowchart for the construction of multigene base editing vectors. The single sgRNA was first inserted into the *Bsa*I site of the target plasmid, which served as a Level 1 vector. The primers containing *Bsa*I enzyme sites and various linkers were designed and used to amplify sgRNA cassettes from Level 1 plasmids. The first target 1 (T_1_) part that was amplified spanned from the guide RNA to the terminator. The last target n-1 (T_n__–__1_) that was amplified spanned from the *pRpmJ* promoter to the guide RNA. Other parts were amplified from the integrated cassettes. The final plasmids were constructed using the Golden Gate Assembly method. **(B)** Editing efficiency assay for target gene conversion using the ABE system. *EW2*, *EW3*, and *EW4* represent three rhizobial genes *SMc00499*, *SMc04218*, and *SMb21346*, respectively. In total, 20 individual colonies were randomly selected for PCR and Sanger sequencing. **(C)** Schematic diagram illustrating the genome structure of the *nodA*, *nodB*, and *nodC* genes. The arrows indicate three sgRNA locations in the genome. **(D)** Editing efficiency assay of *nodB* and *nodC* mutations using the CBE system. **(E,F)** Editing efficiency assay for multigene mutations using the CBE system.

We designed a GGA-compatible tandem multiple cassette (Figure 4A). The Level 1 and Level 2 vectors represent base editing plasmids harboring single sgRNA or multiple sgRNA cassettes in tandem, respectively. We first introduced ABE-*EW234* harboring three sgRNAs cassettes that were previously used to evaluate the editing window of the ABE system. *EW2*, *EW3*, and *EW4* represent *SMc00499*, *SMc04218*, and *SMb21346*, respectively, which are three rhizobial genes. Because the target A of each TA combination in *EW2*, *EW3*, and *EW4* was edited from A to G with an efficiency of 100%, the evaluation of the multigene mutation efficiency was performed by quantifying the transition of target base A to G in each TA combination. Twenty individual colonies were selected for PCR and Sanger sequencing. Eighteen colonies were triple mutants and only two replicates were not edited, indicating an efficiency of nearly 90% (Figure 4B). Interestingly, neither single nor double editing events were observed, which is distinct from previous reports (Xie et al., 2015; Tong et al., 2019).

We next engineered multigene modifications. We used the CBE system to target the *nodA*, *nodB*, and *nodC* genes (Figure 4C). The plasmid CBE-*nodB nodC* containing two sgRNAs was used to calculate the editing efficiency. Sanger sequencing confirmed that both *nodB* and *nodC* were simultaneously edited with an efficiency of 80% (8/10) and that the remaining two strains were not edited (Figure 4D). Consistent with the results from the double sgRNA cassettes, the editing efficiency of the CBEs that simultaneously targeted *nodA*, *nodB*, and *nodC* were slightly decreased. Only 50% of the strains were edited (5/10), and 50% of strains were not edited (Figure 4E). *nodA*, *nodB*, and *nodC* are located on the pSymA plasmid. The distances separating these genes are quite small and thus, genomic DNA disruptions might occur that lower the editing efficiency. To test this hypothesis, we replaced the *nifH* locus with the *nodA* locus because *nifH* is located far from *nodA/B/C*. In this strain, using Sanger sequencing, we confirmed that seven of the ten strains were triple mutants. Thus, although the editing efficiency increased to 70% (Figure 4F), no significant improvement in editing efficiency was observed. Neither CBE-*nodABC* nor CBE-*nifH nodBC* constructs could be forced to acquire single mutants or double mutants, consistent with the aforementioned ABE base editing results. In conclusion, GGA-based multiplex gene base editing tools enabled us to acquire multigene mutants efficiently in one transformation experiment.

### SacB-Based Plasmid Curing After Gene Editing

Plasmid curing is required after target gene modification because the constitutive expression of the Cas9 fusion protein and guide RNAs may be toxic and interfere with host cell viability. Several strategies were employed to cure plasmids after the target gene was successfully mutated (Chen et al., 2017, 2018; Rodrigues et al., 2021). The sucrose-inducible *sacB* gene product from *Bacillus subtilis* is lethal for gram-negative bacteria and has been used as an efficient counter-selectable marker for the construction of suicide vectors in rhizobia (Quandt and Hynes, 1993). The s*acB* was therefore employed for plasmid curing in our experiments (Supplementary Figure 4A). To cure plasmids after editing, three mutated strains [*Sm1021-nodA(Q6R)*] (Supplementary Figure 4B) harboring ABE plasmids were cultured and then streaked on TY plates with 10% (w/v) sucrose. Two individual colonies were picked from each plate and were spread on a solid TY medium containing or lacking neomycin to test whether the plasmids were successfully cured. As expected, all of the selected strains grew normally on the TY plates and no notable colonies were observed on the TY plates containing neomycin (Supplementary Figure 4C).

### Whole-Genome Prediction of Guide RNAs for Gene Inactivation in *S. meliloti*

**FIGURE 5:**
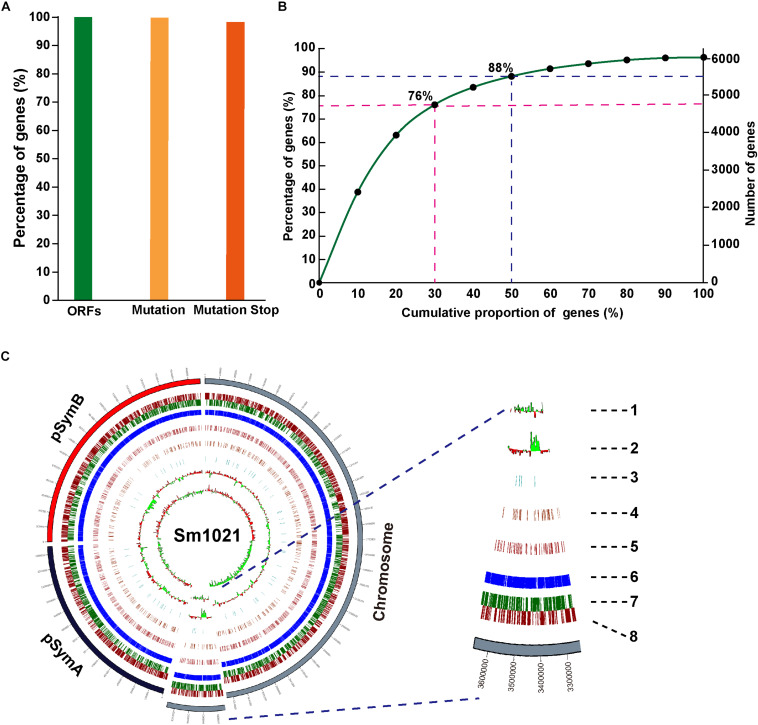
Whole-genome prediction of target loci in *S. meliloti*. **(A)** Proportion of genes containing predicted editable sites for CBE systems. ORFs, the complete open reading frames. Mutation, genes containing predicted editable C-to-T conversion sites located on the sense strand, and genes containing predicted editable G-to-A conversion sites located on the antisense strand. Mutation stop, C-to-T, or G-to-A conversions resulting in premature stop codons. **(B)** Cumulative curve for the distribution of generated premature stop codons inside gene bodies (coding region). **(C)** Circos plot showing the distribution of genes editable by CBE systems in the genome of *S. meliloti*. Concentric circles from the inside to the outside: (1) CG skew; (2) CG content; (3–5) genes with stop mutation sites in 0–30% (3), 30–50% (4), and 50–80% (5) of the gene body; (6) gene annotations; and (7–8) distribution of the genes in reverse (7) and forward (8) strands of the genome.

To gain insights into targetable induced stop codons in the *S. meliloti* genome, we performed a bioinformatics analysis using the iSTOP (induction of STOP codons by CRISPR-mediated base editing) method (Billon et al., 2017). The editing windows of the Cas9n-AID system have a broad range. Therefore, we performed the analysis using the CBE2 system instead of the CBE1 system. Among the predictable 6233 candidate ORFs, almost all of the genes (6173/6233, 99.04%) contained at least one editable C, and 96.21% of genes contained possible iSTOP codons (CAA| Gln, CAG| Gln, CGA| Arg, and TGG| Trp) (Figure 5A). All of the potentially editable stop sites located on the sense or the antisense strand are listed in Supplementary Tables 5, 6. Next, we examined the locations of possible iSTOP codons inside the genes because the distance between iSTOP codons and the start codon has a major impact on the function of the translated premature protein. We found that 63, 76, 88, and 96% of the possibly editable stop sites are located in the first 20, 30, 50, and 80% of the coding region, respectively (Figure 5B). The distribution of targetable iSTOP spacers across the *S. meliloti* genome is shown in Figure 5C. Taken together, the analysis revealed that numerous loci in the *S. meliloti* genome could be inactivated using the CBE2 base editing system.

## Discussion

Symbiotic nitrogen fixation (SNF) is the most efficient form of biological nitrogen fixation (BNF) in terms of supplying nitrogen to plants, and it therefore plays an extremely important role in agriculture. Rhizobia are indispensable symbiotic partners of leguminous plants that facilitate efficient nitrogen fixation. A variety of forward- and reverse-genetic techniques have been well established for genetic manipulations and functional genomics studies during the last four decades, including Tn5-based transposon mutagenesis, plasmid-based allelic replacement, and several site-specific recombinase systems (diCenzo et al., 2019). These gene manipulation systems play a dominate role in the genetic studies of rhizobia. Nevertheless, limitations and drawbacks remain. For instance, multiplexed modifications of gene families could be very time- and labor-consuming using allelic replacement approaches. CRISPR-mediated base editing systems have been widely applied in a variety of microbes because of their simplicity and efficiency (Chen et al., 2018; Gu et al., 2018; Zheng et al., 2018; Tong et al., 2019; Zhang Y. et al., 2020; Rodrigues et al., 2021). *S. meliloti* is an ideal organism for base editing because the genome has a high GC content (62.7%) and thus, “NGG” PAM sites appear frequently in target loci. In this study, we first engineered single-plasmid CRISPR-mediated base editing tools for *S. meliloti* that contain adenosine base editors (ABEs), cytidine base editors (CBEs), and glycosylase base editors (GBEs) that could efficiently catalyze both base transitions (A-to-G, C-to-T) and transversions (C-to-G) for one locus or multiple genes. Meanwhile, due to the simplicity of a one-plasmid system, these tools may be useful for other rhizobial species. Moreover, the application of this technology may be broadened by using native promoters to drive the expression of sgRNA cassettes.

The ABE systems converted A to G at target genomic loci with different efficiencies that ranged from zero to 100%. We found that ABE systems prefer to use “TA” as a substrate relative to distinct “NA” combinations. The TA > CA > AA > GA preference that we observed (Figure 1F) is consistent with previous work (Tong et al., 2019). The CBE systems harboring PmCDA1 could readily catalyze the C-to-T conversion with efficiencies up to 100% (Figure 2C and Supplementary Figure 2). Notably, the CBE systems did not prefer a particular substrate combination. Interestingly, the editing window of both the ABE and CBE systems was seven bases (positions 4–10, with PAM at positions 21–23) and 10 bases (positions 1–8, 12, and 15, with PAM at positions 21–23), respectively, in *S. meliloti* and was, therefore, broader than the five-base window that was reported for other prokaryotes (Chen et al., 2018; Gu et al., 2018; Zheng et al., 2018; Tong et al., 2019; Wang et al., 2020; Zhang Y. et al., 2020; Rodrigues et al., 2021). Considerable effort has been devoted to expanding the editing window of base editors. For instance, engineered variants of Cas9 with alternative PAM preferences have been developed to broaden the targeting scope of base editors (Ma et al., 2019; Miller et al., 2020). This strategy could be incorporated into our system in the future. Recently, dual-deaminase base editors that combine both adenine and cytidine base-editing functions achieved A-to-G and C-to-T conversions at the same site simultaneously (Grunewald et al., 2020; Li et al., 2020; Sakata et al., 2020; Zhang X. et al., 2020). Compared to single base editors, the dual base editors also broadened the range of target DNA sequence modifications. The broader editing window dramatically increased the percentage of targetable induced stop (iSTOP) codons in the genome. In total, 76 and 88% of the possibly editable stop sites were found to be located within the first 30 and 50% of genes in *S. meliloti*, respectively (Figure 5). In addition to inactivating genes, our cytidine base editing tools are useful for investigating the function of specific amino acid residues in particular proteins. For example, the Ji lab used the ABE system to characterize four key residues in a staphylopine/metal complex transporter in *Staphylococcus aureus* (Zhang Y. et al., 2020).

In order to expand the application of base editors, we introduced a GGA-based multigene base editing system for *S. meliloti*. Both ABE and CBE systems efficiently converted target bases with relatively high efficiencies that ranged from 50 to 100% utilizing either two or three sgRNAs (Figure 4). It is possible that the base editors may efficiently edit target sites using more than three sgRNAs. High-throughput methods have become more common for functional genomics research in *S. meliloti*, such as ORFeome and *in vivo* expression technology (IVET) based fusion libraries (diCenzo et al., 2019). Given the high efficiency, high fidelity, and template-independent advantages of base editing, the construction of base editor plasmids is much easier than allelic replacement methods. Indeed, the total percentage of recombinant clones was almost 100% for *ccdB* (Figure 1B), a selectable marker that kills cells lacking recombinant DNA. Therefore, our base editor systems will be more suitable for high-throughput genome engineering in the future.

Off-target effects are a potential challenge for the application of CRISPR-mediated base editors. We systemically evaluated the off-target effects caused by our ABE and CBE systems (Supplementary Tables 1, 2) and observed no detectable off-target events, which is consistent with the negligible level of off-target effects reported previously (Tong et al., 2019; Zhang Y. et al., 2020). Although the efficiency of off-target editing was sufficiently low, we eliminated the possibility of off-target editing by introducing plasmid curing with the sucrose-inducible SacB protein that induces lethality in gram-negative bacteria (Supplementary Figure 4). The plasmid-free cells, serving as new recipients, would be suitable for multiple cycles of gene manipulation.

In summary, the CRISPR-mediated base editing tools can serve as an effective supplement to current techniques for studying rhizobia. The development and application of base editors will accelerate the functional genomics and genome manipulation of rhizobia.

## Data Availability Statement

The raw data supporting the conclusions of this article will be made available by the authors, without undue reservation.

## Author Contributions

LW designed the research and analyzed the results. LW, YX, XW, and JP performed the experiments. LW and DD analyzed the data and wrote the manuscript. DD supervised the project. All authors read and approved the manuscript.

## Conflict of Interest

The authors declare that the research was conducted in the absence of any commercial or financial relationships that could be construed as a potential conflict of interest.

## Footnotes

1 http://www.rgenome.net/be-designer/

2 https://ggtools.neb.com/getset/run.cgi

3 http://www.rgenome.net/cas-offinder/

4 http://circos.ca

## Supplementary Material

Supplementary Figure 1 Cas9 protein expression and the A-to-G conversion of ABE systems in *S. meliloti*. **(A)** ABE fusion protein expression was assayed using an anti-Cas9 antibody with His-tagged Cas9 protein serving as a positive control. The expression of the ABE fusion protein from *pHemA*, *pNeo*, and *pTau* was detectable. Cells possessing an empty vector were used as the negative control. **(B–J)** Comparison of editing efficiencies of ABE systems with various guide RNAs harboring A(s) at different positions in the protospacer. P4–P8 represent the position of A(s) in the protospacer. The protospacer adjacent motif (PAM) spans positions 21–23. The representative sequencing chromatogram for each target locus and the calculated editing efficiencies are shown.

Supplementary Figure 2 Editing window of CBE systems in *S. meliloti*. The editing windows of the CBE2 system were compared by analyzing various guide RNAs harboring Cs at different positions of the protospacer. P1–P8, P12, and P15 indicate the position of C(s) in the protospacer. The protospacer adjacent motif (PAM) is located at positions 21–23. The representative sequencing chromatogram for each target locus and the calculated editing efficiencies are shown.

Supplementary Figure 3 Editing window of GBE systems in *S. meliloti*. **(A)** Schematic diagram of a unique cytidine base editor (CBE). This CBE lacks the Uracil DNA glycosylase inhibitor (UGI) element at the C-terminus of the SpCas9 nickase (Cas9n D10A) that could promote the catalysis of C-to-G transversions. A representative sequencing chromatogram for the target *nodA* is shown **(B)**. **(C–F)** Representative sequencing chromatograms and the calculated editing efficiencies for each target locus. P2–P8 indicates the position of C(s) in the protospacer. The protospacer adjacent motif (PAM) is located at positions 21–23.

Supplementary Figure 4 *SacB*-based plasmid curing after gene editing. **(A)** Schematic flowchart for plasmid curing. Strains containing the *sacB* plasmid were first analyzed for targeted gene mutations. The mutants were grown in a liquid TY medium without antibiotics. Strains that lost plasmids were selected on solid TY medium containing sucrose. The plasmid-free cells are suitable for the next cycle of gene manipulation. **(B)** Three independent mutant strains with *nodA* mutated by an ABE system. The sequencing chromatographs of PCR products are oriented from 5’ to 3’. **(C)** Selection of colonies after plasmid curing. Two individual colonies from each strain were selected from a solid TY/sucrose medium. The antibiotic sensitivity was assayed. No growth was observed in the presence of neomycin.

Supplementary Table 1 Off-target analysis of ABE in *S. meliloti.*

Supplementary Table 2 Off-target analysis of CBE in *S. meliloti.*

Supplementary Table 3 Primers used in this study.

Supplementary Table 4 DNA sequences used in this study.

Data Sheet 1 sgRNAs containing iSTOP targetable sites in forward strands of the genome.

Data Sheet 2 sgRNAs containing iSTOP targetable sites in reverse strands of the genome.
