# Supplementary figures and images for: Highly Efficient CRISPR-Mediated Base Editing in Sinorhizobium meliloti
Source: Front Microbiol. 2021 Jun 18;12:686008. doi: 10.3389/fmicb.2021.686008 (PMC8253261; doi:10.3389/fmicb.2021.686008)

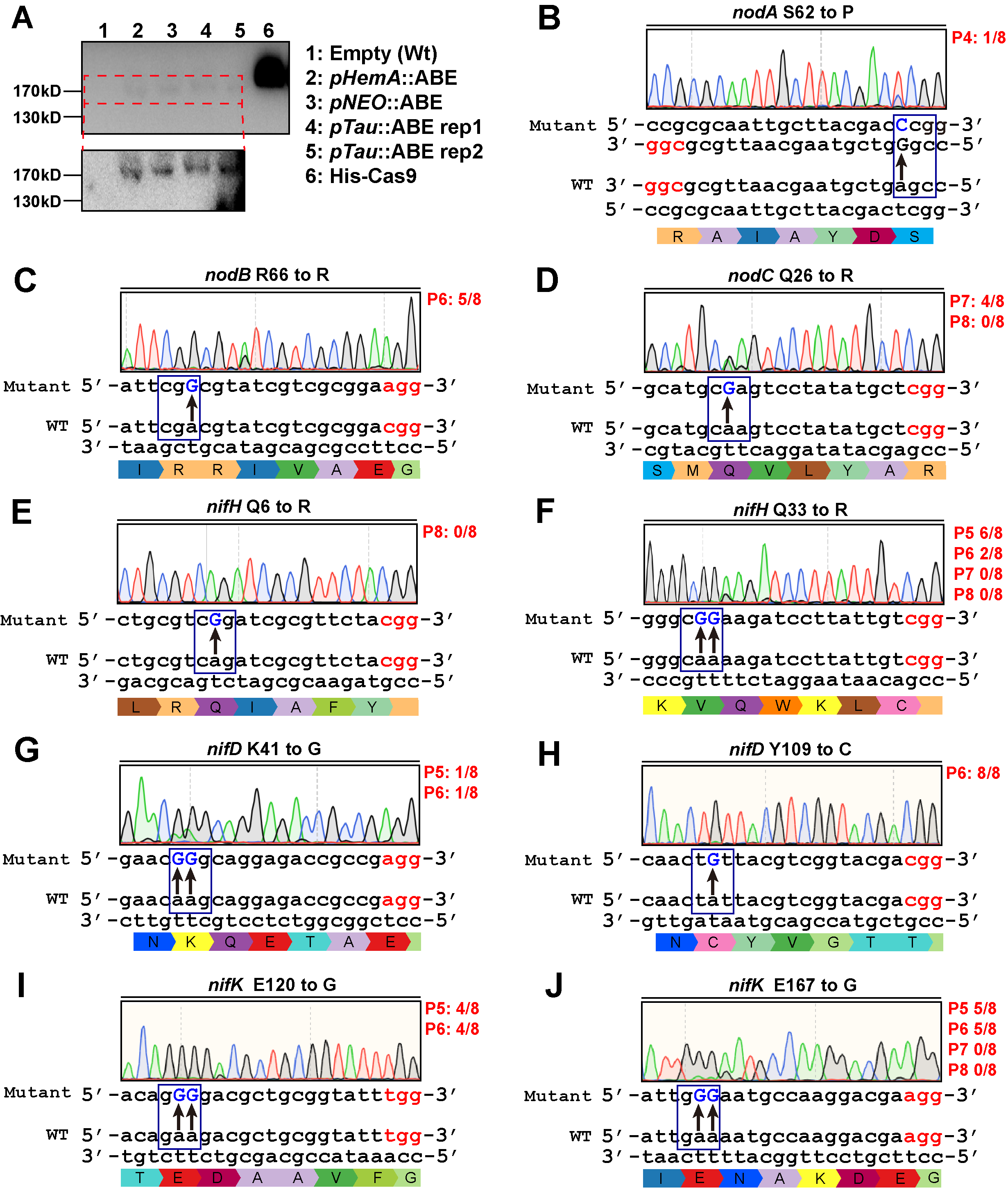

Supplement: Supplementary Figure 1 — Cas9 protein expression and the A-to-G conversion of ABE systems in S. meliloti. (A) ABE fusion protein expression was assayed using an anti-Cas9 antibody with His-tagged Cas9 protein serving as a positive control. The expression of the ABE fusion protein from pHemA, pNeo, and pTau was detectable. Cells possessing an empty vector were used as the negative control. (B–J) Comparison of editing efficiencies of ABE systems with various guide RNAs harboring A(s) at different positions in the protospacer. P4–P8 represent the position of A(s) in the protospacer. The protospacer adjacent motif (PAM) spans positions 21–23. The representative sequencing chromatogram for each target locus and the calculated editing efficiencies are shown. [file Image_1.TIF]

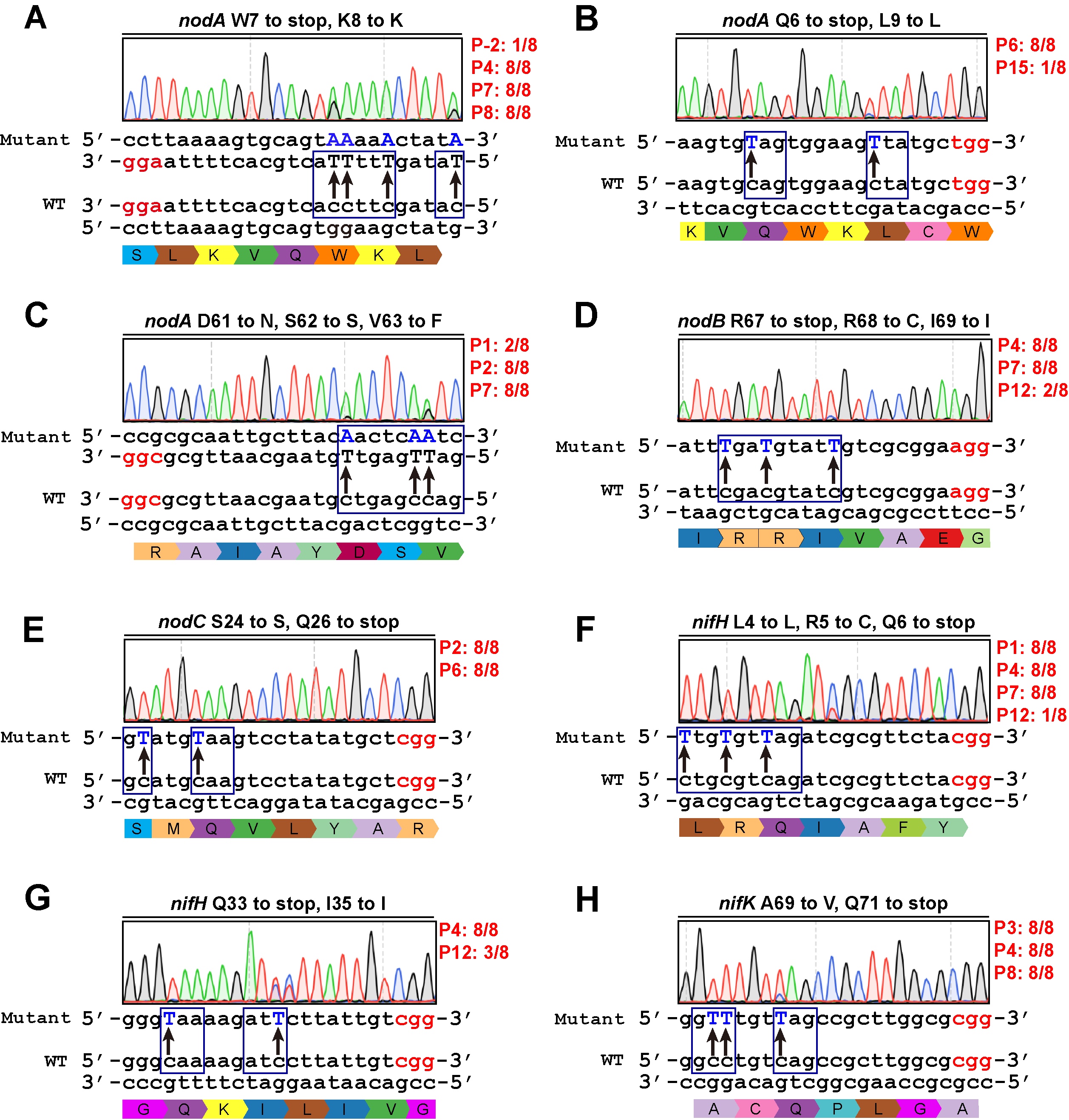

Supplement: Supplementary Figure 2 — Editing window of CBE systems in S. meliloti. The editing windows of the CBE2 system were compared by analyzing various guide RNAs harboring Cs at different positions of the protospacer. P1–P8, P12, and P15 indicate the position of C(s) in the protospacer. The protospacer adjacent motif (PAM) is located at positions 21–23. The representative sequencing chromatogram for each target locus and the calculated editing efficiencies are shown. [file Image_2.TIF]

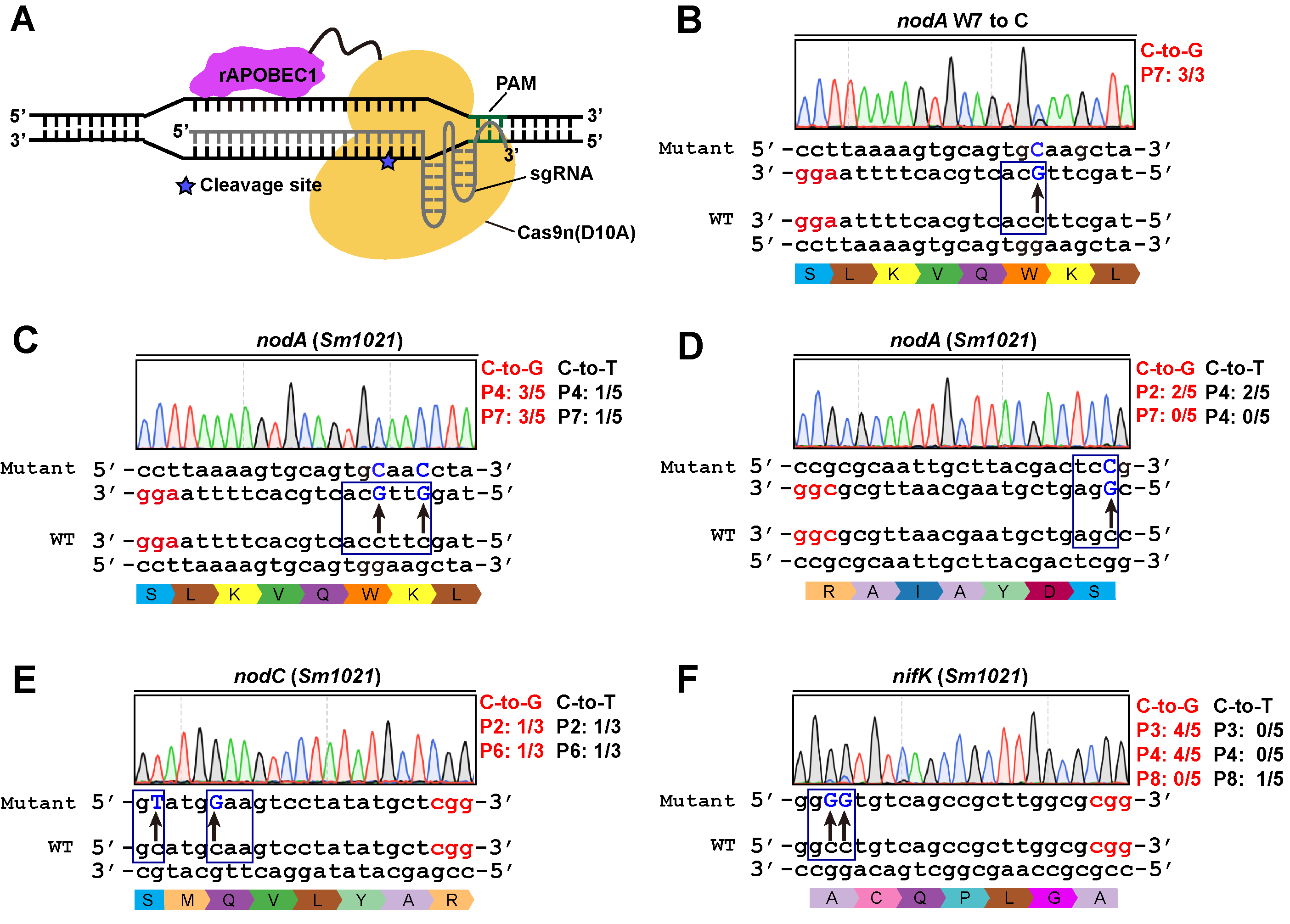

Supplement: Supplementary Figure 3 — Editing window of GBE systems in S. meliloti. (A) Schematic diagram of a unique cytidine base editor (CBE). This CBE lacks the Uracil DNA glycosylase inhibitor (UGI) element at the C-terminus of the SpCas9 nickase (Cas9n D10A) that could promote the catalysis of C-to-G transversions. A representative sequencing chromatogram for the target nodA is shown (B). (C–F) Representative sequencing chromatograms and the calculated editing efficiencies for each target locus. P2–P8 indicates the position of C(s) in the protospacer. The protospacer adjacent motif (PAM) is located at positions 21–23. [file Image_3.TIF]

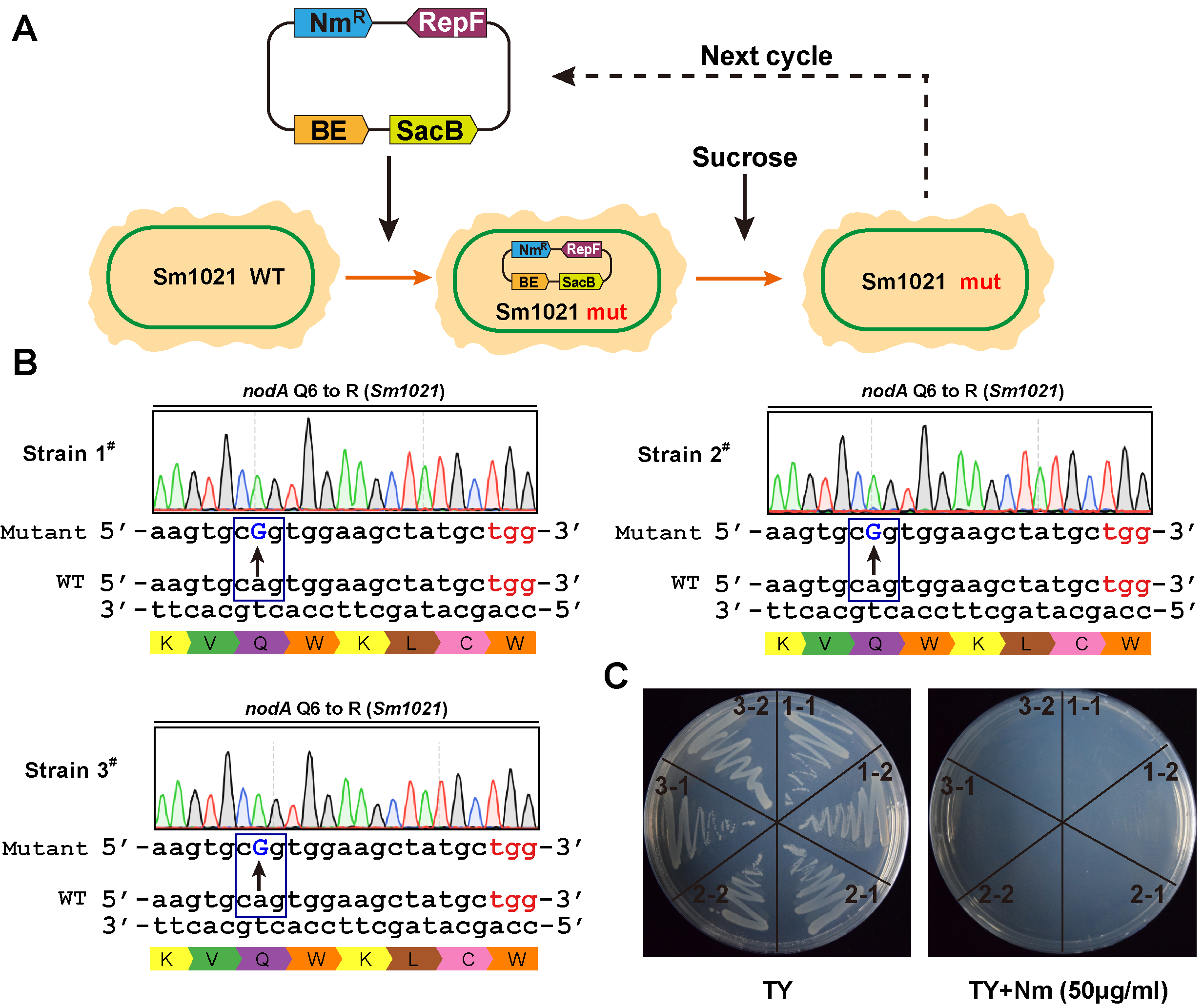

Supplement: Supplementary Figure 4 — SacB-based plasmid curing after gene editing. (A) Schematic flowchart for plasmid curing. Strains containing the sacB plasmid were first analyzed for targeted gene mutations. The mutants were grown in a liquid TY medium without antibiotics. Strains that lost plasmids were selected on solid TY medium containing sucrose. The plasmid-free cells are suitable for the next cycle of gene manipulation. (B) Three independent mutant strains with nodA mutated by an ABE system. The sequencing chromatographs of PCR products are oriented from 5’ to 3’. (C) Selection of colonies after plasmid curing. Two individual colonies from each strain were selected from a solid TY/sucrose medium. The antibiotic sensitivity was assayed. No growth was observed in the presence of neomycin. [file Image_4.TIF]
