# Supplementary material for: Highly Efficient CRISPR-Mediated Base Editing in Sinorhizobium meliloti
Source: Front Microbiol. 2021 Jun 18;12:686008. doi: 10.3389/fmicb.2021.686008 (PMC8253261; doi:10.3389/fmicb.2021.686008)
Supplement: Supplementary Table 1 — Off-target analysis of ABE in S. meliloti. [file Table_1.DOCX]

**Table S1. The off-target analysis of ABE in *S. meliloti***

| **Gene** | **DNA sequence ^(1)^** | **Chromosome ^(2)^** | **Position** | **Mismatch** | **Mutation efficiency ^(3)^** |
| --- | --- | --- | --- | --- | --- |
| on-target: *nodA* | AAGTGCAGTGGAAGCTATGCTGG | NC_003037.1 | 481511 | 0 | 10/10 |
| off1: *SMb20573* | A**cc**T**t**CA**ag**GGAAGCTAT**a**CCGG | NC_003078.1 | 1586372 | 6 | 0/10 |
| off2: *SMc04196* | AAG**c**G**a**A**aa**GGAAGC**g**ATG**g**CGG | NC_003047.1 | 2183944 | 6 | 0/10 |
| off3: *SMa1209* | AA**a**T**c**CAGTGGAAG**ag**AT**c**GCGGG | NC_003037.1 | 664582 | 5 | 0/10 |
| off4: *SMa1779* | AAG**ga**CA**agc**GAAGCTATG**t**CGG | NC_003037.1 | 1008703 | 6 | 0/10 |
| off5: *SMb21087* | **gg**GTGCA**c**T**tc**AAGCT**g**TGCCGG | NC_003078.1 | 730126 | 6 | 0/10 |
| off6: *SMa1394* | AA**cg**GCAGT**t**GAAGC-**g**TGCCGG | NC_003037.1 | 767180 | 4 | 0/10 |
| off7: *SMb20902* | **g**A**a**TGCA-TGGA**c**GCTATG**a**TGG | NC_003078.1 | 1311719 | 4 | 0/10 |
| off8: *SMa2381* | A**c**GTGCA**c**TGGA**g**G**g**TAT-CCGG | NC_003037.1 | 1345137 | 4 | 0/10 |

(1): The top eight potential off-target sites and their genome positions of *S. meliloti* 1021 were predicted by Cas-OFFinder. The mismatched bases are labeled with the thicker lowercase letter and the gaps are represented by a short line. The PAM sequences are underlined.

(2): The *S. meliloti 1021* genomic DNA is consisting of three parts, the chromosome (NC_003047.1) and two symbiotic megaplasmids (pSymA, NC_003037.1 and pSymB, NC_003078.1).

(3): Ten individual colonies genomic DNA was extracted via *EasyPure^®^* Bacteria Genomic DNA Kit (TransGen Biotech Co., LTD) and the mutation efficiency was calculated by PCR-Sanger sequence.
