# Supplementary material for: Highly Efficient CRISPR-Mediated Base Editing in Sinorhizobium meliloti
Source: Front Microbiol. 2021 Jun 18;12:686008. doi: 10.3389/fmicb.2021.686008 (PMC8253261; doi:10.3389/fmicb.2021.686008)
Supplement: Supplementary Table 2 — Off-target analysis of CBE in S. meliloti. [file Table_2.DOCX]

**Table S2. The off-target analysis of CBE in *S. meliloti***

| **Gene** | **DNA sequence ^(1)^** | **Chromosome ^(2)^** | **Position** | **Mismatch** | **Mutation efficiency ^(3)^** |
| --- | --- | --- | --- | --- | --- |
| on-target: *nodA* | TAGCTTCCACTGCACTTTTAAGG | NC_003037.1 | 481517 | 0 | 10/10 |
| off1: *SMa1939* | **c**AGCTTCCAC**cc**CA**aa**TTTACGG | NC_003037.1 | 1102233 | 5 | 0/10 |
| off2: *SMc01779* | T**c**GC**ca**C**gg**CTGCACTTTT**c**CGG | NC_003047.1 | 1323950 | 6 | 0/10 |
| off3: *SMa0667* | **g**A**t**CT**g**C**a**ACTGCACTTT**gc**TGG | NC_003037.1 | 355702 | 6 | 0/10 |
| off4: *SMa0513* | T**t**GCTTCCACATGC**g**CTT**c**TACGG | NC_003037.1 | 277058 | 3 | 0/10 |
| off5: *SMb21069* | T**c**G**g**-TCCACTG**g**ACT**a**TTATGG | NC_003078.1 | 704443 | 4 | 0/10 |
| off6: *SMb20079* | T**g**GC**g**TC**g**ACTGCAC**cg**TT**c**AGG | NC_003078.1 | 89351 | 6 | 0/10 |
| off7: *SMc01321* | T**gc**CTT**t**CACTGC**t**C**c**TTT**g**CGG | NC_003047.1 | 1461594 | 6 | 0/10 |
| off8: *SMb21211* | T**c**GCTTCCA-TGCA**tg**TTT**t**CGG | NC_003078.1 | 964891 | 4 | 0/10 |

(1): The top eight potential off-target sites and their genome positions of *S. meliloti* 1021 were predicted by Cas-OFFinder. The mismatched bases are labeled with the thicker lowercase letter, the gaps are represented by a short line, and the DNA bulges are marked by shadow. The PAM sequences are underlined.

(2): The *S. meliloti 1021* genomic DNA is consisting of three parts, the chromosome (NC_003047.1) and two symbiotic megaplasmids (pSymA, NC_003037.1 and pSymB, NC_003078.1).

(3): Ten individual strains genomic DNA was extracted via *EasyPure^®^* Bacteria Genomic DNA Kit (TransGen Biotech Co., LTD) and the mutation efficiency was calculated by PCR-Sanger sequence.
