# Supplementary material for: Highly Efficient CRISPR-Mediated Base Editing in Sinorhizobium meliloti
Source: Front Microbiol. 2021 Jun 18;12:686008. doi: 10.3389/fmicb.2021.686008 (PMC8253261; doi:10.3389/fmicb.2021.686008)
Supplement: Supplementary Table 3 — Primers used in this study. [file Table_3.DOCX]

**Table S3. Primers used in this study.**

| **Name** | **Sequences (5'-3')** | **Application** |
| --- | --- | --- |
| **Primers for backbone plasmids construction** | | |
| Kan-Esp3I-F | TGCGTCTCATAGTTCAATTAATTATTAGAAAAATTCATCCAGCATC | Cloning |
| Kan-Esp3I-R | TGCGTCTCAGCAATTGTGTCTCAAAATCTCTG | Cloning |
| KX-oligo-F | AGCGGGTACCCGGGGATCCTCTAGA | Cloning |
| KX-oligo-R | GGAGTCTAGAGGATCCCCGGGTACC | Cloning |
| **Primers for Cas9n fusion proteins' promoter assay** | | |
| HemeApro-KpnI-F | CGGGGTACCGACCTGCTTTCCGATATTC | Cloning |
| HemeApro-XhoI-R | GGAGCTCGAGCTCCAGTGCCCTGTCGCC | Cloning |
| pNEO-KpnI-F | CGGGGTACCATTTGTCCTACTCAGGAG | Cloning, also for *pTau* |
| pNEO-SalI-R | ACGCGTCGACTACCATGGCATGCATCG | Cloning |
| pTau-mut-F | CACCGGATTTAGATATCGATACCCCGC | Cloning |
| pTau-mut-R | ATCGATATCTAAATCCGGTGATTGATTGAG | Cloning |
| pTau-BsaI-R | CTGGTCTCCTCGAGTTGTTCTTCCTCCTGTCG | Cloning |
| **Primers for sgRNAs' promoter assay** | | |
| pSigA-XbaI-F | GGAGTCTAGATTTTTCGCCGGAAACGCT | Cloning |
| pSigA-fu-F | ACCATTTCACGAGACCATTGGTCTCAG | Cloning |
| pSigA-fu-R | ATGGTCTCGTGAAATGGTGATTCCCAG | Cloning |
| pRpoN-XbaI-F | GGAGTCTAGACGCCGCCGTCCACGCTTG | Cloning |
| pRpoN-fu-F | CAATTTTTGGGTTCGAGACCATTGGTCTCAG | Cloning |
| pRpoN-fu-R | ATGGTCTCGAACCCAAAAATTGCTTATTAC | Cloning |
| pRpmJ-XbaI-F | GGAGTCTAGAAAATCCCCGGCACGCGGT | Cloning |
| pRpmJ-fu-F | TGCCTGCCTCGAGACCATTGGTCTCAG | Cloning |
| pRpmJ-fu-R | ATGGTCTCGAGGCAGGCACATACCGTG | Cloning |
| pRpsT-XbaI-F | GGAGTCTAGATTTCTCAAATTGCGCGTTG | Cloning |
| pRpsT-fu-F | GCCGCCCTCCGAGACCATTGGTCTCAG | Cloning |
| pRpsT-fu-R | ATGGTCTCGGAGGGCGGCGTATAAAGG | Cloning |
| pTyr-XbaI-F | GGAGTCTAGATTTGCATTTCGCCGTGTTG | Cloning |
| pTyr-fu-F | ACCCGGCGCCGAGACCATTGGTCTCAG | Cloning |
| pTyr-fu-R | ATGGTCTCGGCGCCGGGTATATGACCG | Cloning |
| MssI-sgRNA-R | TGAGTTTAAACTAAGGATCTCCAGGCATC | Cloning |
| **Primers for pKm-HABE, pKm-HCBE, and pKm-HGBE plasmids construction** | | |
| ABE-XhoI-F | CCGCTCGAGATGTCGGAGGTCGAGTTC | Cloning |
| ABE-MssI-R | CTTAGTTTAAACTCAGTCGCCGCCGAGCTG | Cloning |
| CBE-XhoI-F | GACAGGGCACTGGAGCTCGAGATGGACAAGAAGTACAGC | Cloning |
| CBE-MssI-R | GCCGGGGATTTGTTTAAACTCACAGCATCTTGATCTTG | Cloning |
| GBE-XhoI-F | CAGGGCACTGGAGCTCGAGATGTTCGGCGAGTCGTGGAAG | Cloning |
| GBE-MssI-R | GATTTGTTTAAACTCAGCCGCTGCCGCGCGAGAC | Cloning |
| pRpmJ-MssI-F | CTTAGTTTAAACAAATCCCCGGCACGCGGT | Cloning |
| sgRNA-XbaI-R | GGAGTCTAGATACTAGAGTCACACTGGCCC | Cloning |
| ccdB-BsaI-F | TGCCTGCCTGGAGACCGCGGCCGCATTAGGCACC | Cloning |
| ccdB-BsaI-R | CTCTAAAACTGAGACCCTGCAGACTGGCTGTG | Cloning |
| SacB-Gbison-XbaI-F | AGTGTGACTCTAGTATCTAGAGAGAGCGTTCACCGACAA | Cloning |
| SacB-Gbison-XbaI-R | AATTCGGATCCGGAGTAACAGGAGTCCAAGAGCG | Cloning |
| **Primers for single gene editing assay** | | |
| SmNodA-oligo1-F | GCCTTAGCTTCCACTGCACTTTTA | Cloning |
| SmNodA-oligo1-R | AAACTAAAAGTGCAGTGGAAGCTA | Cloning |
| SmNodA-oligo2-F | GCCTAAGTGCAGTGGAAGCTATGC | Cloning |
| SmNodA-oligo2-R | AAACGCATAGCTTCCACTGCACTT | Cloning |
| SmNodA-oligo3-F | GCCTCCGAGTCGTAAGCAATTGCG | Cloning |
| SmNodA-oligo3-R | AAACCGCAATTGCTTACGACTCGG | Cloning |
| SmNodB-oligo1-F | GCCTATTCGACGTATCGTCGCGGA | Cloning |
| SmNodB-oligo1-R | AAACTCCGCGACGATACGTCGAAT | Cloning |
| SmNodC-oligo1-F | GCCTGCATGCAAGTCCTATATGCT | Cloning |
| SmNodC-oligo1-R | AAACAGCATATAGGACTTGCATGC | Cloning |
| SmNifH-oligo1-F | GCCTCTGCGTCAGATCGCGTTCTA | Cloning |
| SmNifH-oligo1-R | AAACTAGAACGCGATCTGACGCAG | Cloning |
| SmNifH-oligo2-F | GCCTGGGCAAAAGATCCTTATTGT | Cloning |
| SmNifH-oligo2-R | AAACACAATAAGGATCTTTTGCCC | Cloning |
| SmNifD-oligo1-F | GCCTGAACAAGCAGGAGACCGCCG | Cloning |
| SmNifD-oligo1-R | AAACCGGCGGTCTCCTGCTTGTTC | Cloning |
| SmNifD-oligo3-F | GCCTCAACTATTACGTCGGTACGA | Cloning |
| SmNifD-oligo3-R | AAACTCGTACCGACGTAATAGTTG | Cloning |
| SmNifK-oligo1-F | GCCTGGCCTGTCAGCCGCTTGGCG | Cloning |
| SmNifK-oligo1-R | AAACCGCCAAGCGGCTGACAGGCC | Cloning |
| SmNifK-oligo3-F | GCCTACAGAAGACGCTGCGGTATT | Cloning |
| SmNifK-oligo3-R | AAACAATACCGCAGCGTCTTCTGT | Cloning |
| SmNifK-oligo4-F | GCCTATTGAAAATGCCAAGGACGA | Cloning |
| SmNifK-oligo4-R | AAACTCGTCCTTGGCATTTTCAAT | Cloning |
| CR-SmNodA-F | TCACAAGTACAGGATGGG | PCR and Sanger sequencing |
| CR-SmNodA-R | TCCCGCAAAGTTGGAGTC | PCR and Sanger sequencing |
| CR-SmNodB-F | ATGAAGCACCTCGATTAC | PCR and Sanger sequencing |
| CR-SmNodB-R | TAAGCGCTCCCGATTCGTC | PCR and Sanger sequencing |
| CR-SmNodC-F | ATGGCGCTTTCCCGTATCG | PCR and Sanger sequencing |
| CR-SmNodC-R | GAGGCAAGCTTGGAGACC | PCR and Sanger sequencing |
| CR-SmNifH-F | CGACTTTTGCACGATCAGC | PCR and Sanger sequencing |
| CR-SmNifH-R | GCCGCCGCATACTACGTCC | PCR and Sanger sequencing |
| CR-SmNifD-F | CTTTGCACGAGAAGCTTATC | PCR and Sanger sequencing |
| CR-SmNifD-R | GAAGCCTTCGCAACGCAC | PCR and Sanger sequencing |
| CR-SmNifK-F | GAAGCGAAACTTCGAACGAC | PCR and Sanger sequencing |
| CR-SmNifK-R | GACATGGCTGCCGACGAATG | PCR and Sanger sequencing |
| **Primers for ABE editing window assay** | | |
| EW1-oligo-F | GCCTAATAGACAAGAGCTTCGGGC | Cloning |
| EW1-oligo-R | AAACGCCCGAAGCTCTTGTCTATT | Cloning |
| EW2-oligo-F | GCCTAGGTAGACAACAGGCCGGGC | Cloning |
| EW2-oligo-R | AAACGCCCGGCCTGTTGTCTACCT | Cloning |
| EW3-oligo-F | GCCTGAAATAGACGGGATTTCCTA | Cloning |
| EW3-oligo-R | AAACTAGGAAATCCCGTCTATTTC | Cloning |
| EW4-oligo-F | GCCTGGCAATAGAGGCGGCGGGCA | Cloning |
| EW4-oligo-R | AAACTGCCCGCCGCCTCTATTGCC | Cloning |
| EW5-oligo-F | GCCTATGCAATAGAGATAGCCGAC | Cloning |
| EW5-oligo-R | AAACGTCGGCTATCTCTATTGCAT | Cloning |
| EW6-oligo-F | GCCTCGGACAATATCTTTCGATCA | Cloning |
| EW6-oligo-R | AAACTGATCGAAAGATATTGTCCG | Cloning |
| EW7-oligo-F | GCCTCTCGACAATAATCCTTCGCC | Cloning |
| EW7-oligo-R | AAACGGCGAAGGATTATTGTCGAG | Cloning |
| CR-ABE-EW1-F | CAGCTGTGTATATTCGCG | PCR and Sanger sequencing |
| CR-ABE-EW1-R | GCCGAGCATGACGTTTTC | PCR and Sanger sequencing |
| CR-ABE-EW2-F | ACAAGAAGAACGTTTGGG | PCR and Sanger sequencing |
| CR-ABE-EW2-R | TGTACTGATGAAAGCCGCC | PCR and Sanger sequencing |
| CR-ABE-EW3-F | TTCCACGAGGCAGAGTTC | PCR and Sanger sequencing |
| CR-ABE-EW3-R | CGAAGGGATGAGCGATGC | PCR and Sanger sequencing |
| CR-ABE-EW4-F | GCCAACGACCGCAAGAAAG | PCR and Sanger sequencing |
| CR-ABE-EW4-R | CGAAGAATGTCAGTAGGCG | PCR and Sanger sequencing |
| CR-ABE-EW5-F | GCATCGACACAAGCATTG | PCR and Sanger sequencing |
| CR-ABE-EW5-R | CGCCGCGGTAATGATTCTC | PCR and Sanger sequencing |
| CR-ABE-EW6-F | GGCAAGAACATAACCATCG | PCR and Sanger sequencing |
| CR-ABE-EW6-R | TCAGGCAAAGCTCCTTCGC | PCR and Sanger sequencing |
| CR-ABE-EW7-F | GGCGATCCTTTCCGAGAC | PCR and Sanger sequencing |
| CR-ABE-EW7-R | ATCGGCGATGGTGGTTTC | PCR and Sanger sequencing |
| **Primers for ABE off-target assay** | | |
| SmNodA2-off1-F | TGTGATTGAATTTGCGGCTAG | PCR and Sanger sequencing |
| SmNodA2-off1-R | CCTTCGTGGACCATATCG | PCR and Sanger sequencing |
| SmNodA2-off2-F | GAGTGCCCTCTTACGTGATC | PCR and Sanger sequencing |
| SmNodA2-off2-R | GATGACACCTTCAGCCGG | PCR and Sanger sequencing |
| SmNodA2-off3-F | TGCATCGAACCAGGCATG | PCR and Sanger sequencing |
| SmNodA2-off3-R | CAATCGAAGGAGCGCTGC | PCR and Sanger sequencing |
| SmNodA2-off4-F | TAAGGTTTTGGCAGAGAC | PCR and Sanger sequencing |
| SmNodA2-off4-R | GTTTCATGCAATCCGGCG | PCR and Sanger sequencing |
| SmNodA2-off5-F | TCGCCGCTCCTCACTATG | PCR and Sanger sequencing |
| SmNodA2-off5-R | TTGGCGAACTCTCGGGCGATG | PCR and Sanger sequencing |
| SmNodA2-off6-F | CCGTCACAAGTATTTTCATGG | PCR and Sanger sequencing |
| SmNodA2-off6-R | TGCACAAGCAAGCGGCGTG | PCR and Sanger sequencing |
| SmNodA2-off7-F | TCAACGCGCTGCGGGAATG | PCR and Sanger sequencing |
| SmNodA2-off7-R | GATTGCGTCGGAGTCCTG | PCR and Sanger sequencing |
| SmNodA2-off8-F | CTTTATGGCACGGATCAAG | PCR and Sanger sequencing |
| SmNodA2-off8-R | CCTGATACCGGCGATATTG | PCR and Sanger sequencing |
| **Primers for CBE off-target assay** | | |
| SmNodA1-off1-F | CCGAGATCGTAGTGAATATCC | PCR and Sanger sequencing |
| SmNodA1-off1-R | CTGGTACCCGAACAGCATG | PCR and Sanger sequencing |
| SmNodA1-off2-F | CGCCGAAACGTGCAGTTGC | PCR and Sanger sequencing |
| SmNodA1-off2-R | CGACGAAAGCACCCGCAAAC | PCR and Sanger sequencing |
| SmNodA1-off3-F | CTGCCACTTTATAGGAAGG | PCR and Sanger sequencing |
| SmNodA1-off3-R | TATTGTCACCTGCCACGG | PCR and Sanger sequencing |
| SmNodA1-off4-F | ATGAAGGCGATCGTCATCC | PCR and Sanger sequencing |
| SmNodA1-off4-R | ATCGACAGCGTGCCGATC | PCR and Sanger sequencing |
| SmNodA1-off5-F | CCGCGGCATAGAAATCCTTC | PCR and Sanger sequencing |
| SmNodA1-off5-R | ACTCCGTGACCCAGATGG | PCR and Sanger sequencing |
| SmNodA1-off6-F | TACAGCGACAAGGCGGCG | PCR and Sanger sequencing |
| SmNodA1-off6-R | AAGGCGGTGGTCGGGTTTG | PCR and Sanger sequencing |
| SmNodA1-off7-F | ACCGTTCAGGATGTCGATG | PCR and Sanger sequencing |
| SmNodA1-off7-R | GTGATGACGACCGGGATCG | PCR and Sanger sequencing |
| SmNodA1-off8-F | AAGGTTCAATCCGTCCGGAC | PCR and Sanger sequencing |
| SmNodA1-off8-R | GTTGCAAAGCGGGCTGTTC | PCR and Sanger sequencing |
| **Primers for multiplex gene editing assay** | | |
| GGA-linker2F | CATTGGTCTCAACTCAAATCCCCGGCACGCGGT | Cloning, common primers |
| GGA-linker2R | CAATGGTCTCGGAGTATTTGTCCTACTCAGGAG | Cloning, common primers |
| GGA-linker3F | CATTGGTCTCACATTAAATCCCCGGCACGCGGT | Cloning, common primers |
| GGA-linker3R | CAATGGTCTCGAATGATTTGTCCTACTCAGGAG | Cloning, common primers |
| ABE-EW234-linker1F | CATTGGTCTCAGCCTAGGTAGACAACAGGCCGGGC | Cloning |
| ABE-EW234-linker4R | CAATGGTCTCGAAACTGCCCGCCGCCTCTATTGCC | Cloning |
| CBE-NodABC-linker1F | CATTGGTCTCAGCCTTAGCTTCCACTGCACTTTTA | Cloning |
| CBE-NodABC-linker4R | CAATGGTCTCGAAACAGCATATAGGACTTGCATGC | Cloning |
| CBE-NodBC-linker1F | CATTGGTCTCAGCCTATTCGACGTATCGTCGCGGA | Cloning |
| CBE-NifH NodBC-linker1F | CATTGGTCTCAGCCTGGGCAAAAGATCCTTATTGTG | Cloning |
