# Supplementary material for: Highly Efficient CRISPR-Mediated Base Editing in Sinorhizobium meliloti
Source: Front Microbiol. 2021 Jun 18;12:686008. doi: 10.3389/fmicb.2021.686008 (PMC8253261; doi:10.3389/fmicb.2021.686008)
Supplement: Supplementary Table 4 — DNA sequences used in this study. [file Table_4.DOCX]

**Table S4. DNA sequences used in this study.**

| ***pHemA* promoter** |
| --- |
| GACCTGCTTTCCGATATTCATGCAAGCGCCGCCTATCGCGCCAATCTCGTCAAGGTGATGACGAAACGCGCCGTGGCGGCCGCCTGACAGGCCAACTTTGGTATCGAAAAAGGGCGGCAGCGGCCGTCCTTTTTCCGCAATTGCTTGACTTCGATCGATGTTCGGGAGAATGAAGTTTTGCCAGCCATTTTGGAATGGTTCAAAACTGGGCACTCGGACTCGGTGTGATGCCGCGCCTGATTACGGCGGAAGGTTTGAAAATGTGTCGTCATTTTCCATCGCCTTTCCTGCTTGCCAGGCACGACACGCGCCGCGCAGAATGACCGAGGTCTGGGGCCGAGAGGCCTTTTGCCGCAGCCCGCGCGTCGGAGCCTGTCCGGGGTTGACCACTGATCGCTTTGAAGGAAGAAAGGCGACAGGGCACTGGAG |
| ***pNeo* promoter** |
| ATTTGTCCTACTCAGGAGAGCGTTCACCGACAAACAACAGATAAAACGAAAGGCCCAGTCTTTCGACTGAGCCTTTCGTTTTATTTGATGCCTGCCGAATTCGGATCCGGAGAGGCCTGAATCGCCCCATCATCCAGCCAGAAAGTGAGGGAGCCACGGTTGATGAGAGCTTTGTTGTAGGTGGACCAGTTGGTGATTTTGAACTTTTGCTTTGCCACGGAACGGTCTGCGTTGTCGGGAAGATGCGTGATCTGATCCTTCAACTCAGCAAAAGTTCGATTTATTCAACAAAGCGACGTTGTGTCTCAAAATCTCTGATGTTACATTGCACAAGATAAAAATATATCATCATGAACAATAAAACTGTCTGCTTACATAAACAGTAATACAAGGGGTGTTATGAGCCATATTCAACGGGAAACGTCTTGCTCGAGATCTATCGATGCATGCCATGGTA |
| ***pTau* promoter** |
| ATTTGTCCTACTCAGGAGAGCGTTCACCGACAAACAACAGATAAAACGAAAGGCCCAGTCTTTCGACTGAGCCTTTCGTTTTATTTGATGCCTGCCGAATTCGGATCCGGAGGGCTTTACTAAGCTGATCCGGTGGATGACCTTTTGAATGACCTTTAATAGATTATATTACTAATTAATTGGGGACCCTAGAGGTCCCCTTTTTTATTTTAAAAATTTTTTCACAAAACGGTTTACAAGCATAAAGCTTGCTCGATCAATCACCGGATTTAGATATCGATACCCCGCTCCGCGATCCGCGCCGCAGTCAGGGCCACGCCGTCGGCTATGCGGTTGCGCGCGATCGACGAGTAGCCCATGCGGAAGAAGCGGCATGGGCCGTCGTCCTTCGGGAAGAAGGGCGACCCGGATTCGATCAGCACGCCGTCCTTTCGCAAAGCGTTCATCAGCAAGTCCGCATCCAGACCTTCCGGCCCCTCGATCCAGAAGGACGTGCCGCCGAAAGCCGCAGATCCGGCGATCTCCAATCCGGCATCCCGGAGGGCAGCGTCCATGATGATGTGGCGGCGATGATACTCCTCGCGCATGCGGTGCAGCACGGCATCATAGTGTCCGAGCGCCAGGAAATAGGCGGCCGTTCTCTGGAGGTGCCCCGGCGGATGCCGCAACATCAAGGCCCGTAGTGCCCGCGCCTCCCGGATCACGGCAGCCGGCGCCACCAGATAGCCCAGACGCAGGCCGGGAAACAACGACTTGGAGAAACTGCCGATGTAGAACACCCGGCCGCAGCGGTCGAAGGCCTTGAGGGCCGGGGAGGGGGGCGCCAGAAAGCTCATCTCGAACTCGTAATCGTCCTCGACGATTATGAAGTCCTTGGAGGCGGCCGCCTCGAGCAAGGCCGCCCGCCGTTCGATCGGCATCGTCGCGCCGGTCGGCGAATGATGGCTGGGCGTGACGAACACGGCATCGACCTCTTCTGGAAGCGCGGCGGGCGGCAGGCCGTCCTTGTCCACGTCGACGAAGGTGATCCCGGCGCCGCTCAAGCGAAGCGATGCACTCATGTCGGGATGGCAGGGGTTCTCGCAGACCGCATGGGCGCCCTGACGCAACAGGAGCTGGATGACGATCCAGAGGGCGTTCTGCGCGCCCACCGTCACCAGGATCTCGTCGGGGTTCGCGCGGATGCCGCGGCCCGGCAATGTGCGCGAGCAGATATAGTTGACAAGCTGGATGTCGTCCGCCGCCGCGAAGTCGCCCGCCATCAGCTCGAAATCCTCGCGCGCCAGCGCCCGTCGCGCGCAGTCGCGCCAGGCCGTGAGGTCGAACAGCGACGGGTCCATCTGACCGTATAGGAACGGATAAGGGTAGGTTCGCCAGTCGAGGGGCTTACGCATCTGCTTGGCCACGATGAAGGTCGAGCGCAACTTGGCCGACCAGTCGATGGCATCGGCCGCAGCATGGGCGGCCTGCATCGCGATGGCTGTCGTTGGGGGCTTGCCGGCCACACGGTAGCCGCTCCGGTTCGCCGCCTCCACATAGCCCTGGGAAGCGAGTTCCTGATAGGCGAGCGTTACGGTGATCCGCGAAATATTCAGATAGGCCGCAAGCTTGCGGCTGGAGGGCAATTGGGCGCCCGGCGCCAGGCGGCCCGACAATACGGCCGAAACGATCGTTTCGCGGATCTGCGCCTGCAGGCCCGTTTGGCTGGCCCGGTCAAGGAAAAAGATCGTTTCGGACACGGTCGAGCCCGCCATTGTTCACCTCGCCCAACTGGACTCATCTAACTTCCTATCTGGATATAAGGCAATCCGAAGCGTGGGCCTATTTCTATTCAGCAAGCAAAGACCGTCATTCAATGTACGGCGCGACCCCGCAAAAAGCGGGGCGACATAACCAAAGAGGGTAACGACAGGAGGAAGAACAA |
| ***pSigA* promoter** |
| TTTTTCGCCGGAAACGCTTGACGGGATGAAAAATTCTGGGAATCACCATTTCA |
| ***pRpoN* promoter** |
| CGCCGCCGTCCACGCTTGACCAAATTCCAGTAATAAGCAATTTTTGGGTT |
| ***pRpsT* promoter** |
| TTTCTCAAATTGCGCGTTGACGTGCGGGCGGTTTCCCTTTATACGCCGCCCTC |
| ***pTyr* promoter** |
| TTTGCATTTCGCCGTGTTGACAGGACGAATGCAGGCGGTCATATACCCGGCGC |
| ***pRpmJ* promoter** |
| AAATCCCCGGCACGCGGTTGACACGATTGGGTCGACACGGTATGTGCCTGCCT |
| ***ccdB-Cm: sgRNA*** |
| GGAGACCGCGGCCGCATTAGGCACCCCAGGCTTTACACTTTATGCTTCCGGCTCGTATAATGTGTGGATTTTGAGTTAGGATCCGTCGAGATTTTCAGGAGCTAAGGAAGCTAAAATGGAGAAAAAAATCACTGGATATACCACCGTTGATATATCCCAATGGCATCGTAAAGAACATTTTGAGGCATTTCAGTCAGTTGCTCAATGTACCTATAACCAGACCGTTCAGCTGGATATTACGGCCTTTTTAAAGACCGTAAAGAAAAATAAGCACAAGTTTTATCCGGCCTTTATTCACATTCTTGCCCGCCTGATGAATGCTCATCCGGAATTCCGTATGGCAATGAAAGACGGTGAGCTGGTGATATGGGATAGTGTTCACCCTTGTTACACCGTTTTCCATGAGCAAACTGAAACGTTTTCATCGCTCTGGAGTGAATACCACGACGATTTCCGGCAGTTTCTACACATATATTCGCAAGATGTGGCGTGTTACGGTGAAAACCTGGCCTATTTCCCTAAAGGGTTTATTGAGAATATGTTTTTCGTCTCAGCCAATCCCTGGGTGAGTTTCACCAGTTTTGATTTAAACGTGGCCAATATGGACAACTTCTTCGCCCCCGTTTTCACCATGGGCAAATATTATACGCAAGGCGACAAGGTGCTGATGCCGCTGGCGATTCAGGTTCATCATGCCGTTTGTGATGGCTTCCATGTCGGCAGAATGCTTAATGAATTACAACAGTACTGCGATGAGTGGCAGGGCGGGGCGTAAAGATCTGGATCCGGCTTACTAAAAGCCAGATAACAGTATGCGTATTTGCGCGCTGATTTTTGCGGTATAAGAATATATACTGATATGTATACCCGAAGTATGTCAAAAAGAGGTATGCTATGAAGCAGCGTATTACAGTGACAGTTGACAGCGACAGCTATCAGTTGCTCAAGGCATATATGATGTCAATATCTCCGGTCTGGTAAGCACAACCATGCAGAATGAAGCCCGTCGTCTGCGTGCCGAACGCTGGAAAGCGGAAAATCAGGAAGGGATGGCTGAGGTCGCCCGGTTTATTGAAATGAACGGCTCTTTTGCTGACGAGAACAGGGGCTGGTGAAATGCAGTTTAAGGTTTACACCTATAAAAGAGAGAGCCGTTATCGTCTGTTTGTGGATGTACAGAGTGATATTATTGACACGCCCGGGCGACGGATGGTGATCCCCCTGGCCAGTGCACGTCTGCTGTCAGATAAAGTCTCCCGTGAACTTTACCCGGTGGTGCATATCGGGGATGAAAGCTGGCGCATGATGACCACCGATATGGCCAGTGTGCCGGTGTCCGTTATCGGGGAAGAAGTGGCTGATCTCAGCCACCGCGAAAATGACATCAAAAACGCCATTAACCTGATGTTCTGGGGAATATAAATGTCAGGCTCCCTTATACACAGCCAGTCTGCAGGGTCTCAGTTTTAGAGCTAGAAATAGCAAGTTAAAATAAGGCTAGTCCGTTATCAACTTGAAAAAGTGGCACCGAGTCGGTGCTTTTTTTGAGATCTGTCCATACCCATGGCCCGGGCCAGTGTGACTCTAGTA |
| **ABE base editors** |
| ATGTCGGAGGTCGAGTTCAGCCACGAGTATTGGATGCGCCATGCCCTGACCCTCGCCAAGCGCGCGTGGGACGAGCGCGAGGTCCCGGTCGGCGCCGTCCTGGTCCACAACAACCGCGTCATCGGCGAGGGCTGGAACCGCCCGATCGGCCGCCATGACCCGACCGCCCATGCCGAGATCATGGCCCTGCGCCAGGGCGGCCTCGTCATGCAGAACTATCGCCTCATCGACGCGACCCTGTACGTCACCCTGGAGCCGTGCGTCATGTGCGCGGGCGCCATGATCCACTCGCGCATCGGCCGCGTCGTCTTCGGCGCCCGCGACGCCAAGACGGGCGCCGCGGGCAGCCTGATGGACGTCCTCCACCATCCGGGCATGAACCATCGCGTCGAGATCACCGAGGGCATCCTGGCGGACGAGTGCGCCGCGCTGCTCTCGGACTTCTTCCGCATGCGTCGCCAGGAGATCAAGGCCCAGAAGAAGGCGCAGTCGAGCACCGACTCGGGCGGCTCGTCGGGCGGCTCGTCGGGTAGCGAGACGCCGGGCACGTCGGAGAGCGCGACGCCGGAGAGCTCGGGCGGCTCGTCGGGCGGCTCGTCGGAAGTGGAGTTCAGCCACGAGTACTGGATGCGGCACGCCCTGACGCTGGCCAAGCGCGCGCGCGACGAACGGGAGGTCCCGGTCGGCGCGGTCCTGGTCCTGAATAATCGGGTGATCGGCGAAGGCTGGAACCGCGCCATCGGCCTGCATGATCCGACCGCGCACGCCGAAATCATGGCCCTCCGGCAGGGCGGCCTCGTCATGCAAAACTATCGCCTGATCGACGCCACCCTCTACGTCACCTTCGAACCGTGCGTGATGTGCGCGGGCGCGATGATCCATTCGCGCATCGGCCGGGTGGTCTTCGGCGTCCGCAATGCGAAGACGGGCGCGGCCGGCAGCCTGATGGATGTGCTCCACTACCCGGGCATGAATCACCGGGTGGAAATCACCGAAGGCATCCTGGCCGATGAATGCGCCGCGCTGCTCTGCTACTTCTTCCGCATGCCGCGCCAGGTCTTCAACGCGCAGAAGAAGGCGCAGAGCTCGACGGATTCGGGCGGCTCGTCGGGCGGCTCGTCGGGGTCGGAGACGCCTGGCACGTCGGAGAGCGCCACGCCGGAGTCCTCGGGCGGCTCGTCGGGCGGCAGCATCGGAGACAAGAAGTACAGCATCGGCCTGGCCATCGGCACGAACAGCGTCGGCTGGGCGGTCATCACCGACGAGTATAAGGTCCCGTCGAAGAAGTTCAAGGTCCTGGGCAACACCGACCGCCACAGCATCAAGAAGAACCTCATCGGCGCCCTGCTGTTCGACAGCGGCGAGACGGCGGAGGCCACCCGCCTGAAGCGCACCGCCCGTCGCCGCTACACGCGTCGCAAGAACCGCATCTGCTATCTCCAGGAGATCTTCTCGAACGAGATGGCCAAGGTCGACGACTCGTTCTTCCATCGCCTGGAGGAGAGCTTCCTCGTCGAAGAAGATAAGAAGCACGAGCGCCATCCGATCTTCGGCAACATCGTCGACGAGGTCGCGTATCACGAGAAGTACCCGACGATCTATCATCTCCGCAAGAAGCTGGTCGACAGCACCGACAAGGCCGACCTGCGCCTCATCTACCTGGCCCTCGCGCACATGATCAAGTTCCGCGGCCATTTCCTGATCGAGGGCGACCTCAACCCGGACAACTCGGACGTCGACAAGCTCTTCATCCAGCTGGTCCAGACGTATAACCAGCTGTTCGAGGAGAACCCGATCAACGCCAGCGGCGTCGACGCCAAGGCCATCCTCTCGGCCCGCCTGAGCAAGTCGCGCCGCCTGGAGAACCTGATCGCCCAGCTCCCGGGCGAGAAGAAGAACGGCCTCTTCGGCAACCTCATCGCCCTGAGCCTGGGCCTGACCCCGAACTTCAAGAGCAACTTCGACCTGGCCGAGGACGCGAAGCTCCAGCTGTCGAAGGACACCTACGACGACGACCTCGACAACCTGCTCGCCCAGATCGGCGACCAGTATGCGGACCTCTTCCTGGCCGCGAAGAACCTGTCGGACGCGATCCTGCTCAGCGACATCCTCCGCGTCAACACCGAGATCACGAAGGCCCCGCTGTCGGCGAGCATGATCAAGCGCTACGACGAGCACCATCAGGACCTCACGCTCCTGAAGGCCCTGGTCCGCCAGCAGCTCCCGGAGAAGTATAAGGAGATCTTCTTCGACCAGAGCAAGAACGGCTATGCGGGCTACATCGACGGCGGCGCAAGCCAGGAAGAGTTCTACAAGTTCATCAAGCCGATCCTGGAGAAGATGGACGGCACGGAGGAGCTGCTCGTCAAGCTCAACCGCGAGGACCTGCTCCGCAAGCAGCGCACCTTCGACAACGGCTCGATCCCGCACCAGATCCATCTCGGCGAGCTGCATGCGATCCTGCGTCGCCAGGAAGACTTCTATCCGTTCCTCAAGGACAACCGCGAGAAGATCGAGAAGATCCTGACGTTCCGCATCCCGTATTATGTCGGCCCGCTGGCACGCGGCAACTCGCGCTTCGCGTGGATGACCCGCAAGAGCGAGGAGACGATCACGCCGTGGAACTTCGAGGAAGTCGTCGACAAGGGCGCCAGCGCCCAGAGCTTCATCGAGCGCATGACGAACTTCGACAAGAACCTGCCGAACGAGAAGGTCCTCCCGAAGCACAGCCTGCTCTATGAGTACTTCACCGTCTACAACGAGCTGACGAAGGTCAAGTATGTCACCGAAGGCATGCGCAAGCCGGCCTTCCTCAGCGGCGAGCAGAAGAAGGCGATCGTCGACCTGCTCTTCAAGACCAACCGCAAGGTCACGGTCAAGCAGCTGAAGGAAGACTACTTCAAGAAGATCGAGTGCTTCGACTCGGTCGAGATCAGCGGCGTCGAGGACCGCTTCAACGCCAGCCTGGGCACCTATCATGACCTGCTCAAGATCATCAAGGACAAGGACTTCCTCGACAACGAGGAGAACGAGGATATCCTGGAGGACATCGTCCTGACCCTCACGCTGTTCGAGGACCGCGAGATGATCGAGGAGCGCCTGAAGACCTATGCCCACCTCTTCGACGACAAGGTCATGAAGCAGCTGAAGCGTCGCCGCTACACCGGCTGGGGCCGCCTGTCGCGCAAGCTCATCAACGGCATCCGCGACAAGCAGTCGGGCAAGACCATCCTCGACTTCCTGAAGAGCGACGGCTTCGCCAACCGCAACTTCATGCAGCTGATCCACGACGACAGCCTCACGTTCAAGGAAGACATCCAGAAGGCCCAGGTCAGCGGCCAGGGCGACAGCCTGCACGAACACATCGCCAACCTGGCCGGCTCGCCGGCCATCAAGAAGGGCATCCTGCAGACCGTCAAGGTCGTCGACGAGCTCGTCAAGGTCATGGGCCGCCATAAGCCGGAGAACATCGTCATCGAGATGGCCCGCGAGAACCAGACCACGCAGAAGGGCCAGAAGAACAGCCGCGAGCGCATGAAGCGCATCGAGGAAGGCATCAAGGAGCTGGGCTCGCAGATCCTCAAGGAGCACCCGGTCGAGAACACCCAGCTGCAGAACGAGAAGCTCTATCTGTATTACCTCCAGAACGGCCGCGACATGTACGTCGACCAGGAGCTGGACATCAACCGCCTCAGCGACTATGACGTCGACCATATCGTCCCGCAGTCGTTCCTGAAGGACGACAGCATCGACAACAAGGTCCTCACGCGCTCGGACAAGAACCGCGGCAAGTCGGACAACGTCCCGAGCGAGGAAGTCGTCAAGAAGATGAAGAACTACTGGCGCCAGCTGCTCAACGCCAAGCTGATCACCCAGCGCAAGTTCGACAACCTCACCAAGGCGGAGCGCGGCGGCCTCTCGGAGCTGGACAAGGCGGGCTTCATCAAGCGCCAGCTGGTCGAGACGCGCCAGATCACGAAGCACGTCGCGCAGATCCTCGACTCGCGCATGAACACCAAGTATGACGAGAACGACAAGCTGATCCGCGAGGTCAAGGTCATCACGCTGAAGTCGAAGCTCGTCAGCGACTTCCGCAAGGACTTCCAGTTCTATAAGGTCCGCGAGATCAACAACTATCACCATGCCCATGACGCCTACCTCAACGCGGTCGTCGGCACCGCGCTGATCAAGAAGTACCCGAAGCTGGAGTCGGAGTTCGTCTATGGCGACTACAAGGTCTATGACGTCCGCAAGATGATCGCCAAGAGCGAGCAGGAGATCGGCAAGGCCACGGCGAAGTACTTCTTCTATTCGAACATCATGAACTTCTTCAAGACCGAGATCACCCTCGCCAACGGCGAGATCCGCAAGCGCCCGCTCATCGAGACGAACGGCGAGACGGGCGAGATCGTCTGGGACAAGGGCCGCGACTTCGCCACGGTCCGCAAGGTCCTCAGCATGCCGCAGGTCAACATCGTCAAGAAGACCGAGGTCCAGACGGGCGGCTTCTCGAAGGAGAGCATCCTGCCGAAGCGCAACTCGGACAAGCTCATCGCCCGCAAGAAGGACTGGGACCCGAAGAAGTATGGTGGCTTCGACAGCCCGACGGTCGCCTACTCGGTCCTGGTCGTCGCGAAGGTCGAGAAGGGCAAGTCGAAGAAGCTCAAGAGCGTCAAGGAGCTGCTCGGCATCACGATCATGGAGCGCTCGTCGTTCGAGAAGAACCCGATCGACTTCCTGGAGGCGAAGGGCTATAAGGAAGTCAAGAAGGACCTGATCATCAAGCTCCCGAAGTACTCGCTCTTCGAGCTGGAGAACGGCCGCAAGCGCATGCTCGCTAGCGCCGGCGAGCTCCAGAAGGGCAACGAGCTCGCCCTGCCGTCGAAGTACGTCAACTTCCTCTATCTGGCGTCGCACTACGAGAAGCTCAAGGGCAGCCCGGAGGACAACGAGCAGAAGCAGCTGTTCGTCGAGCAGCACAAGCATTATCTCGACGAGATCATCGAGCAGATCTCGGAGTTCAGCAAGCGCGTCATCCTGGCCGACGCGAACCTGGACAAGGTCCTCAGCGCCTACAACAAGCATCGCGACAAGCCGATCCGCGAGCAGGCGGAGAACATCATCCATCTCTTCACGCTGACCAACCTGGGCGCCCCGGCCGCGTTCAAGTATTTCGACACCACGATCGACCGCAAGCGCTACACCTCGACGAAGGAAGTCCTGGACGCGACGCTCATCCATCAGTCGATCACCGGCCTGTACGAGACGCGCATCGACCTGAGCCAGCTCGGCGGCGACTGA |
| **CBE base editors** |
| ATGGACAAGAAGTACAGCATCGGCCTGGCCATCGGCACGAACAGCGTCGGCTGGGCGGTCATCACCGACGAGTATAAGGTCCCGTCGAAGAAGTTCAAGGTCCTGGGCAACACCGACCGCCACAGCATCAAGAAGAACCTCATCGGCGCCCTGCTGTTCGACAGCGGCGAGACGGCGGAGGCCACCCGCCTGAAGCGCACCGCCCGTCGCCGCTACACGCGTCGCAAGAACCGCATCTGCTATCTCCAGGAGATCTTCTCGAACGAGATGGCCAAGGTCGACGACTCGTTCTTCCATCGCCTGGAGGAGAGCTTCCTCGTCGAAGAAGATAAGAAGCACGAGCGCCATCCGATCTTCGGCAACATCGTCGACGAGGTCGCGTATCACGAGAAGTACCCGACGATCTATCATCTCCGCAAGAAGCTGGTCGACAGCACCGACAAGGCCGACCTGCGCCTCATCTACCTGGCCCTCGCGCACATGATCAAGTTCCGCGGCCATTTCCTGATCGAGGGCGACCTCAACCCGGACAACTCGGACGTCGACAAGCTCTTCATCCAGCTGGTCCAGACGTATAACCAGCTGTTCGAGGAGAACCCGATCAACGCCAGCGGCGTCGACGCCAAGGCCATCCTCTCGGCCCGCCTGAGCAAGTCGCGCCGCCTGGAGAACCTGATCGCCCAGCTCCCGGGCGAGAAGAAGAACGGCCTCTTCGGCAACCTCATCGCCCTGAGCCTGGGCCTGACCCCGAACTTCAAGAGCAACTTCGACCTGGCCGAGGACGCGAAGCTCCAGCTGTCGAAGGACACCTACGACGACGACCTCGACAACCTGCTCGCCCAGATCGGCGACCAGTATGCGGACCTCTTCCTGGCCGCGAAGAACCTGTCGGACGCGATCCTGCTCAGCGACATCCTCCGCGTCAACACCGAGATCACGAAGGCCCCGCTGTCGGCGAGCATGATCAAGCGCTACGACGAGCACCATCAGGACCTCACGCTCCTGAAGGCCCTGGTCCGCCAGCAGCTCCCGGAGAAGTATAAGGAGATCTTCTTCGACCAGAGCAAGAACGGCTATGCGGGCTACATCGACGGCGGCGCAAGCCAGGAAGAGTTCTACAAGTTCATCAAGCCGATCCTGGAGAAGATGGACGGCACGGAGGAGCTGCTCGTCAAGCTCAACCGCGAGGACCTGCTCCGCAAGCAGCGCACCTTCGACAACGGCTCGATCCCGCACCAGATCCATCTCGGCGAGCTGCATGCGATCCTGCGTCGCCAGGAAGACTTCTATCCGTTCCTCAAGGACAACCGCGAGAAGATCGAGAAGATCCTGACGTTCCGCATCCCGTATTATGTCGGCCCGCTGGCACGCGGCAACTCGCGCTTCGCGTGGATGACCCGCAAGAGCGAGGAGACGATCACGCCGTGGAACTTCGAGGAAGTCGTCGACAAGGGCGCCAGCGCCCAGAGCTTCATCGAGCGCATGACGAACTTCGACAAGAACCTGCCGAACGAGAAGGTCCTCCCGAAGCACAGCCTGCTCTATGAGTACTTCACCGTCTACAACGAGCTGACGAAGGTCAAGTATGTCACCGAAGGCATGCGCAAGCCGGCCTTCCTCAGCGGCGAGCAGAAGAAGGCGATCGTCGACCTGCTCTTCAAGACCAACCGCAAGGTCACGGTCAAGCAGCTGAAGGAAGACTACTTCAAGAAGATCGAGTGCTTCGACTCGGTCGAGATCAGCGGCGTCGAGGACCGCTTCAACGCCAGCCTGGGCACCTATCATGACCTGCTCAAGATCATCAAGGACAAGGACTTCCTCGACAACGAGGAGAACGAGGATATCCTGGAGGACATCGTCCTGACCCTCACGCTGTTCGAGGACCGCGAGATGATCGAGGAGCGCCTGAAGACCTATGCCCACCTCTTCGACGACAAGGTCATGAAGCAGCTGAAGCGTCGCCGCTACACCGGCTGGGGCCGCCTGTCGCGCAAGCTCATCAACGGCATCCGCGACAAGCAGTCGGGCAAGACCATCCTCGACTTCCTGAAGAGCGACGGCTTCGCCAACCGCAACTTCATGCAGCTGATCCACGACGACAGCCTCACGTTCAAGGAAGACATCCAGAAGGCCCAGGTCAGCGGCCAGGGCGACAGCCTGCACGAACACATCGCCAACCTGGCCGGCTCGCCGGCCATCAAGAAGGGCATCCTGCAGACCGTCAAGGTCGTCGACGAGCTCGTCAAGGTCATGGGCCGCCATAAGCCGGAGAACATCGTCATCGAGATGGCCCGCGAGAACCAGACCACGCAGAAGGGCCAGAAGAACAGCCGCGAGCGCATGAAGCGCATCGAGGAAGGCATCAAGGAGCTGGGCTCGCAGATCCTCAAGGAGCACCCGGTCGAGAACACCCAGCTGCAGAACGAGAAGCTCTATCTGTATTACCTCCAGAACGGCCGCGACATGTACGTCGACCAGGAGCTGGACATCAACCGCCTCAGCGACTATGACGTCGACCATATCGTCCCGCAGTCGTTCCTGAAGGACGACAGCATCGACAACAAGGTCCTCACGCGCTCGGACAAGAACCGCGGCAAGTCGGACAACGTCCCGAGCGAGGAAGTCGTCAAGAAGATGAAGAACTACTGGCGCCAGCTGCTCAACGCCAAGCTGATCACCCAGCGCAAGTTCGACAACCTCACCAAGGCGGAGCGCGGCGGCCTCTCGGAGCTGGACAAGGCGGGCTTCATCAAGCGCCAGCTGGTCGAGACGCGCCAGATCACGAAGCACGTCGCGCAGATCCTCGACTCGCGCATGAACACCAAGTATGACGAGAACGACAAGCTGATCCGCGAGGTCAAGGTCATCACGCTGAAGTCGAAGCTCGTCAGCGACTTCCGCAAGGACTTCCAGTTCTATAAGGTCCGCGAGATCAACAACTATCACCATGCCCATGACGCCTACCTCAACGCGGTCGTCGGCACCGCGCTGATCAAGAAGTACCCGAAGCTGGAGTCGGAGTTCGTCTATGGCGACTACAAGGTCTATGACGTCCGCAAGATGATCGCCAAGAGCGAGCAGGAGATCGGCAAGGCCACGGCGAAGTACTTCTTCTATTCGAACATCATGAACTTCTTCAAGACCGAGATCACCCTCGCCAACGGCGAGATCCGCAAGCGCCCGCTCATCGAGACGAACGGCGAGACGGGCGAGATCGTCTGGGACAAGGGCCGCGACTTCGCCACGGTCCGCAAGGTCCTCAGCATGCCGCAGGTCAACATCGTCAAGAAGACCGAGGTCCAGACGGGCGGCTTCTCGAAGGAGAGCATCCTGCCGAAGCGCAACTCGGACAAGCTCATCGCCCGCAAGAAGGACTGGGACCCGAAGAAGTATGGTGGCTTCGACAGCCCGACGGTCGCCTACTCGGTCCTGGTCGTCGCGAAGGTCGAGAAGGGCAAGTCGAAGAAGCTCAAGAGCGTCAAGGAGCTGCTCGGCATCACGATCATGGAGCGCTCGTCGTTCGAGAAGAACCCGATCGACTTCCTGGAGGCGAAGGGCTATAAGGAAGTCAAGAAGGACCTGATCATCAAGCTCCCGAAGTACTCGCTCTTCGAGCTGGAGAACGGCCGCAAGCGCATGCTCGCTAGCGCCGGCGAGCTCCAGAAGGGCAACGAGCTGGCGCTCCCGTCGAAGTATGTCAACTTCCTGTACCTCGCCTCGCACTATGAGAAGCTGAAGGGCAGCCCGGAGGACAACGAGCAGAAGCAGCTCTTCGTCGAGCAGCACAAGCATTACCTGGACGAGATCATCGAGCAGATCTCGGAGTTCAGCAAGCGCGTCATCCTCGCCGACGCGAACCTCGACAAGGTCCTGTCGGCCTATAACAAGCATCGCGACAAGCCGATCCGCGAGCAGGCGGAGAACATCATCCATCTGTTCACGCTCACCAACCTGGGCGCCCCGGCCGCGTTCAAGTACTTCGACACCACGATCGACCGCAAGCGCTATACCAGCACGAAGGAAGTCCTGGACGCGACCCTGATCCACCAGTCGATCACCGGCCTCTACGAGACGCGCATCGACCTGAGCCAGCTGGGCGGCGACGGTGGCGGCGGCTCGGGCGGCGGCGGCTCGGCCGAGTATGTCCGCGCGCTGTTCGACTTCAACGGCAACGACGAAGAAGATCTGCCGTTCAAGAAGGGCGACATCCTCCGCATCCGCGACAAGCCGGAGGAGCAGTGGTGGAACGCGGAGGACAGCGAGGGCAAGCGCGGCATGATCCCGGTCCCGTATGTCGAGAAGTACTCGGGCAGCCGCATGACCGACGCGGAGTATGTCCGCATCCACGAGAAGCTGGACATCTACACGTTCAAGAAGCAGTTCTTCAACAACAAGAAGTCGGTCAGCCATCGCTGCTATGTCCTCTTCGAGCTCAAGCGCCGCGGCGAGCGTCGCGCCTGCTTCTGGGGCTACGCGGTCAACAAGCCGCAGAGCGGCACCGAGCGCGGCATCCACGCCGAGATCTTCAGCATCCGCAAGGTCGAGGAGTATCTCCGCGACAACCCGGGCCAGTTCACGATCAACTGGTATTCGTCGTGGTCGCCGTGCGCGGACTGCGCCGAGAAGATCCTGGAGTGGTATAACCAGGAGCTCCGCGGCAACGGCCATACCCTGAAGATCTGGGCCTGCAAGCTCTATTACGAGAAGAACGCGCGCAACCAGATCGGCCTGTGGAACCTCCGCGACAACGGCGTCGGCCTGAACGTCATGGTCAGCGAGCACTACCAGTGCTGCCGCAAGATCTTCATCCAGTCGAGCCATAACCAGCTCAACGAGAACCGCTGGCTGGAGAAGACCCTGAAGCGCGCGGAGAAGCGCCGCAGCGAGCTGAGCATCATGATCCAGGTCAAGATCCTCCACACCACGAAGAGCCCGGCCGTCTCGCGCGGCAGCGGCGGCAGCACCAACCTCTCGGACATCATCGAGAAGGAGACGGGCAAGCAGCTGGTCATCCAGGAGAGCATCCTGATGCTCCCGGAGGAAGTCGAGGAAGTCATCGGCAACAAGCCGGAGTCGGACATCCTGGTCCATACCGCGTACGACGAGTCGACGGACGAGAACGTCATGCTGCTCACGAGCGACGCCCCGGAGTATAAGCCGTGGGCGCTCGTCATCCAGGACAGCAACGGCGAGAACAAGATCAAGATGCTGTGA |
| **GBE base editors** |
| ATGTTCGGCGAGTCGTGGAAGAAGCACCTCAGCGGCGAGTTCGGCAAGCCGTATTTCATCAAGCTGATGGGCTTCGTCGCCGAGGAGCGCAAGCACTATACCGTCTATCCGCCGCCGCACCAGGTCTTCACCTGGACCCAGATGTGCGACATCAAGGACGTCAAGGTCGTCATCCTCGGCCAGGACCCGTATCATGGCCCGAACCAGGCCCATGGCCTGTGCTTCAGCGTCCAGCGCCCGGTCCCGCCGCCGCCGTCGCTGGAGAACATCTACAAGGAGCTGAGCACGGACATCGAGGACTTCGTCCATCCGGGCCATGGCGACCTCAGCGGCTGGGCCAAGCAGGGCGTCCTGCTCCTGAACGCCGTCCTCACGGTCCGCGCCCACCAGGCCAACAGCCATAAGGAGCGCGGCTGGGAGCAGTTCACCGACGCCGTCGTCTCGTGGCTCAACCAGAACAGCAACGGCCTGGTCTTCCTCCTGTGGGGCTCGTATGCCCAGAAGAAGGGCTCGGCCATCGACCGCAAGCGCCACCATGTCCTGCAGACGGCCCACCCGTCGCCGCTGTCGGTCTACCGCGGCTTCTTCGGCTGCCGCCATTTCTCGAAGACGAACGAGCTCCTGCAGAAGAGCGGCAAGAAGCCGATCGACTGGAAGGAGCTCAGCGGCTCGGAGACGCCGGGCACGGAGATGGACAAGAAGTACAGCATCGGCCTGGCCATCGGCACGAACAGCGTCGGCTGGGCGGTCATCACCGACGAGTATAAGGTCCCGTCGAAGAAGTTCAAGGTCCTGGGCAACACCGACCGCCACAGCATCAAGAAGAACCTCATCGGCGCCCTGCTGTTCGACAGCGGCGAGACGGCGGAGGCCACCCGCCTGAAGCGCACCGCCCGTCGCCGCTACACGCGTCGCAAGAACCGCATCTGCTATCTCCAGGAGATCTTCTCGAACGAGATGGCCAAGGTCGACGACTCGTTCTTCCATCGCCTGGAGGAGAGCTTCCTCGTCGAAGAAGATAAGAAGCACGAGCGCCATCCGATCTTCGGCAACATCGTCGACGAGGTCGCGTATCACGAGAAGTACCCGACGATCTATCATCTCCGCAAGAAGCTGGTCGACAGCACCGACAAGGCCGACCTGCGCCTCATCTACCTGGCCCTCGCGCACATGATCAAGTTCCGCGGCCATTTCCTGATCGAGGGCGACCTCAACCCGGACAACTCGGACGTCGACAAGCTCTTCATCCAGCTGGTCCAGACGTATAACCAGCTGTTCGAGGAGAACCCGATCAACGCCAGCGGCGTCGACGCCAAGGCCATCCTCTCGGCCCGCCTGAGCAAGTCGCGCCGCCTGGAGAACCTGATCGCCCAGCTCCCGGGCGAGAAGAAGAACGGCCTCTTCGGCAACCTCATCGCCCTGAGCCTGGGCCTGACCCCGAACTTCAAGAGCAACTTCGACCTGGCCGAGGACGCGAAGCTCCAGCTGTCGAAGGACACCTACGACGACGACCTCGACAACCTGCTCGCCCAGATCGGCGACCAGTATGCGGACCTCTTCCTGGCCGCGAAGAACCTGTCGGACGCGATCCTGCTCAGCGACATCCTCCGCGTCAACACCGAGATCACGAAGGCCCCGCTGTCGGCGAGCATGATCAAGCGCTACGACGAGCACCATCAGGACCTCACGCTCCTGAAGGCCCTGGTCCGCCAGCAGCTCCCGGAGAAGTATAAGGAGATCTTCTTCGACCAGAGCAAGAACGGCTATGCGGGCTACATCGACGGCGGCGCAAGCCAGGAAGAGTTCTACAAGTTCATCAAGCCGATCCTGGAGAAGATGGACGGCACGGAGGAGCTGCTCGTCAAGCTCAACCGCGAGGACCTGCTCCGCAAGCAGCGCACCTTCGACAACGGCTCGATCCCGCACCAGATCCATCTCGGCGAGCTGCATGCGATCCTGCGTCGCCAGGAAGACTTCTATCCGTTCCTCAAGGACAACCGCGAGAAGATCGAGAAGATCCTGACGTTCCGCATCCCGTATTATGTCGGCCCGCTGGCACGCGGCAACTCGCGCTTCGCGTGGATGACCCGCAAGAGCGAGGAGACGATCACGCCGTGGAACTTCGAGGAAGTCGTCGACAAGGGCGCCAGCGCCCAGAGCTTCATCGAGCGCATGACGAACTTCGACAAGAACCTGCCGAACGAGAAGGTCCTCCCGAAGCACAGCCTGCTCTATGAGTACTTCACCGTCTACAACGAGCTGACGAAGGTCAAGTATGTCACCGAAGGCATGCGCAAGCCGGCCTTCCTCAGCGGCGAGCAGAAGAAGGCGATCGTCGACCTGCTCTTCAAGACCAACCGCAAGGTCACGGTCAAGCAGCTGAAGGAAGACTACTTCAAGAAGATCGAGTGCTTCGACTCGGTCGAGATCAGCGGCGTCGAGGACCGCTTCAACGCCAGCCTGGGCACCTATCATGACCTGCTCAAGATCATCAAGGACAAGGACTTCCTCGACAACGAGGAGAACGAGGATATCCTGGAGGACATCGTCCTGACCCTCACGCTGTTCGAGGACCGCGAGATGATCGAGGAGCGCCTGAAGACCTATGCCCACCTCTTCGACGACAAGGTCATGAAGCAGCTGAAGCGTCGCCGCTACACCGGCTGGGGCCGCCTGTCGCGCAAGCTCATCAACGGCATCCGCGACAAGCAGTCGGGCAAGACCATCCTCGACTTCCTGAAGAGCGACGGCTTCGCCAACCGCAACTTCATGCAGCTGATCCACGACGACAGCCTCACGTTCAAGGAAGACATCCAGAAGGCCCAGGTCAGCGGCCAGGGCGACAGCCTGCACGAACACATCGCCAACCTGGCCGGCTCGCCGGCCATCAAGAAGGGCATCCTGCAGACCGTCAAGGTCGTCGACGAGCTCGTCAAGGTCATGGGCCGCCATAAGCCGGAGAACATCGTCATCGAGATGGCCCGCGAGAACCAGACCACGCAGAAGGGCCAGAAGAACAGCCGCGAGCGCATGAAGCGCATCGAGGAAGGCATCAAGGAGCTGGGCTCGCAGATCCTCAAGGAGCACCCGGTCGAGAACACCCAGCTGCAGAACGAGAAGCTCTATCTGTATTACCTCCAGAACGGCCGCGACATGTACGTCGACCAGGAGCTGGACATCAACCGCCTCAGCGACTATGACGTCGACCATATCGTCCCGCAGTCGTTCCTGAAGGACGACAGCATCGACAACAAGGTCCTCACGCGCTCGGACAAGAACCGCGGCAAGTCGGACAACGTCCCGAGCGAGGAAGTCGTCAAGAAGATGAAGAACTACTGGCGCCAGCTGCTCAACGCCAAGCTGATCACCCAGCGCAAGTTCGACAACCTCACCAAGGCGGAGCGCGGCGGCCTCTCGGAGCTGGACAAGGCGGGCTTCATCAAGCGCCAGCTGGTCGAGACGCGCCAGATCACGAAGCACGTCGCGCAGATCCTCGACTCGCGCATGAACACCAAGTATGACGAGAACGACAAGCTGATCCGCGAGGTCAAGGTCATCACGCTGAAGTCGAAGCTCGTCAGCGACTTCCGCAAGGACTTCCAGTTCTATAAGGTCCGCGAGATCAACAACTATCACCATGCCCATGACGCCTACCTCAACGCGGTCGTCGGCACCGCGCTGATCAAGAAGTACCCGAAGCTGGAGTCGGAGTTCGTCTATGGCGACTACAAGGTCTATGACGTCCGCAAGATGATCGCCAAGAGCGAGCAGGAGATCGGCAAGGCCACGGCGAAGTACTTCTTCTATTCGAACATCATGAACTTCTTCAAGACCGAGATCACCCTCGCCAACGGCGAGATCCGCAAGCGCCCGCTCATCGAGACGAACGGCGAGACGGGCGAGATCGTCTGGGACAAGGGCCGCGACTTCGCCACGGTCCGCAAGGTCCTCAGCATGCCGCAGGTCAACATCGTCAAGAAGACCGAGGTCCAGACGGGCGGCTTCTCGAAGGAGAGCATCCTGCCGAAGCGCAACTCGGACAAGCTCATCGCCCGCAAGAAGGACTGGGACCCGAAGAAGTATGGTGGCTTCGACAGCCCGACGGTCGCCTACTCGGTCCTGGTCGTCGCGAAGGTCGAGAAGGGCAAGTCGAAGAAGCTCAAGAGCGTCAAGGAGCTGCTCGGCATCACGATCATGGAGCGCTCGTCGTTCGAGAAGAACCCGATCGACTTCCTGGAGGCGAAGGGCTATAAGGAAGTCAAGAAGGACCTGATCATCAAGCTCCCGAAGTACTCGCTCTTCGAGCTGGAGAACGGCCGCAAGCGCATGCTCGCTAGCGCCGGCGAGCTCCAGAAGGGCAACGAGCTGGCGCTCCCGTCGAAGTATGTCAACTTCCTGTACCTCGCCTCGCACTATGAGAAGCTGAAGGGCAGCCCGGAGGACAACGAGCAGAAGCAGCTCTTCGTCGAGCAGCACAAGCATTACCTGGACGAGATCATCGAGCAGATCTCGGAGTTCAGCAAGCGCGTCATCCTCGCCGACGCGAACCTCGACAAGGTCCTGTCGGCCTATAACAAGCATCGCGACAAGCCGATCCGCGAGCAGGCGGAGAACATCATCCATCTGTTCACGCTCACCAACCTGGGCGCCCCGGCCGCGTTCAAGTACTTCGACACCACGATCGACCGCAAGCGCTATACCAGCACGAAGGAAGTCCTGGACGCGACCCTGATCCACCAGTCGATCACCGGCCTCTACGAGACGCGCATCGACCTGAGCCAGCTGGGCGGCGACGGTGGCGGCGGCTCGGGCGGCGGCGGCTCGGCCGAGTATGTCCGCGCGCTGTTCGACTTCAACGGCAACGACGAAGAAGATCTGCCGTTCAAGAAGGGCGACATCCTCCGCATCCGCGACAAGCCGGAGGAGCAGTGGTGGAACGCGGAGGACAGCGAGGGCAAGCGCGGCATGATCCCGGTCCCGTATGTCGAGAAGTACTCGGGCAGCCGCATGACCGACGCGGAGTATGTCCGCATCCACGAGAAGCTGGACATCTACACGTTCAAGAAGCAGTTCTTCAACAACAAGAAGTCGGTCAGCCATCGCTGCTATGTCCTCTTCGAGCTCAAGCGCCGCGGCGAGCGTCGCGCCTGCTTCTGGGGCTACGCGGTCAACAAGCCGCAGAGCGGCACCGAGCGCGGCATCCACGCCGAGATCTTCAGCATCCGCAAGGTCGAGGAGTATCTCCGCGACAACCCGGGCCAGTTCACGATCAACTGGTATTCGTCGTGGTCGCCGTGCGCGGACTGCGCCGAGAAGATCCTGGAGTGGTATAACCAGGAGCTCCGCGGCAACGGCCATACCCTGAAGATCTGGGCCTGCAAGCTCTATTACGAGAAGAACGCGCGCAACCAGATCGGCCTGTGGAACCTCCGCGACAACGGCGTCGGCCTGAACGTCATGGTCAGCGAGCACTACCAGTGCTGCCGCAAGATCTTCATCCAGTCGAGCCATAACCAGCTCAACGAGAACCGCTGGCTGGAGAAGACCCTGAAGCGCGCGGAGAAGCGCCGCAGCGAGCTGAGCATCATGATCCAGGTCAAGATCCTCCACACCACGAAGAGCCCGGCCGTCTCGCGCGGCAGCGGCTGA |
| ***sacB*** |
| GAGAGCGTTCACCGACAAACAACAGATAAAACGAAAGGCCCAGTCTTTCGACTGAGCCTTTCGTTTTATTTGCACATATACCTGCCGTTCACTATTATTTAGTGAAATGAGATATTATGATATTTTCTGAATTGTGATTAAAAAGGCAACTTTATGCCCATGCAACAGAAACTATAAAAAATACAGAGAATGAAAAGAAACAGATAGATTTTTTAGTTCTTTAGGCCCGTAGTCTGCAAATCCTTTTATGATTTTCTATCAAACAAAAGAGGAAAATAGACCAGTTGCAATCCAAACGAGAGTCTAATAGAATGAGGTCGAAAAGTAAATCGCGCGGGTTTGTTACTGATAAAGCAGGCAAGACCTAAAATGTGTAAAGGGCAAAGTGTATACTTTGGCGTCACCCCTTACATATTTTAGGTCTTTTTTTATTGTGCGTAACTAACTTGCCATCTTCAAACAGGAGGGCTGGAAGAAGCAGACCGCTAACACAGTACATAAAAAAGGAGACATGAACGATGAACATCAAAAAGTTTGCAAAACAAGCAACAGTATTAACCTTTACTACCGCACTGCTGGCAGGAGGCGCAACTCAAGCGTTTGCGAAAGAAACGAACCAAAAGCCATATAAGGAAACATACGGCATTTCCCATATTACACGCCATGATATGCTGCAAATCCCTGAACAGCAAAAAAATGAAAAATATCAAGTTTCTGAATTTGATTCGTCCACAATTAAAAATATCTCTTCTGCAAAAGGCCTGGACGTTTGGGACAGCTGGCCATTACAAAACGCTGACGGCACTGTCGCAAACTATCACGGCTACCACATCGTCTTTGCATTAGCCGGAGATCCTAAAAATGCGGATGACACATCGATTTACATGTTCTATCAAAAAGTCGGCGAAACTTCTATTGACAGCTGGAAAAACGCTGGCCGCGTCTTTAAAGACAGCGACAAATTCGATGCAAATGATTCTATCCTAAAAGACCAAACACAAGAATGGTCAGGTTCAGCCACATTTACATCTGACGGAAAAATCCGTTTATTCTACACTGATTTCTCCGGTAAACATTACGGCAAACAAACACTGACAACTGCACAAGTTAACGTATCAGCATCAGACAGCTCTTTGAACATCAACGGTGTAGAGGATTATAAATCAATCTTTGACGGTGACGGAAAAACGTATCAAAATGTACAGCAGTTCATCGATGAAGGCAACTACAGCTCAGGCGACAACCATACGCTGAGAGATCCTCACTACGTAGAAGATAAAGGCCACAAATACTTAGTATTTGAAGCAAACACTGGAACTGAAGATGGCTACCAAGGCGAAGAATCTTTATTTAACAAAGCATACTATGGCAAAAGCACATCATTCTTCCGTCAAGAAAGTCAAAAACTTCTGCAAAGCGATAAAAAACGCACGGCTGAGTTAGCAAACGGCGCTCTCGGTATGATTGAGCTAAACGATGATTACACACTGAAAAAAGTGATGAAACCGCTGATTGCATCTAACACAGTAACAGATGAAATTGAACGCGCGAACGTCTTTAAAATGAACGGCAAATGGTATCTGTTCACTGACTCCCGCGGATCAAAAATGACGATTGACGGCATTACGTCTAACGATATTTACATGCTTGGTTATGTTTCTAATTCTTTAACTGGCCCATACAAGCCGCTGAACAAAACTGGCCTTGTGTTAAAAATGGATCTTGATCCTAACGATGTAACCTTTACTTACTCACACTTCGCTGTACCTCAAGCGAAAGGAAACAATGTCGTGATTACAAGCTATATGACAAACAGAGGATTCTACGCAGACAAACAATCAACGTTTGCGCCGAGCTTCCTGCTGAACATCAAAGGCAAGAAAACATCTGTTGTCAAAGACAGCATCCTTGAACAAGGACAATTAACAGTTAACAAATAAAAACGCAAAAGAAAATGCCGATGGGTACGTACCCGCTCTTGGACTCCTGTT |
